# Temporal changes of ^137^Cs concentrations in the Far Eastern Seas: partitioning of ^137^Cs between overlying waters and sediments

**DOI:** 10.1038/s41598-023-49083-4

**Published:** 2023-12-27

**Authors:** Katsumi Hirose, Pavel P. Povinec

**Affiliations:** 1 Laboratory for Environmental Research at Mount Fuji, Okubo, Shinjyuku-Ku, Tokyo, Japan; 2 Department of Nuclear Physics and Biophysics, Comenius University, Bratislava, Slovakia

**Keywords:** Biogeochemistry, Environmental sciences, Ocean sciences

## Abstract

Deep-ocean sediments, similarly to seawater, are important reservoirs of ^137^Cs, an anthropogenic radionuclide with a relatively long half-live found in the Earth system. To better understand the geochemical behaviour of ^137^Cs in the ocean, we examined the temporal changes of ^137^Cs activity concentrations in the overlying waters and in sediments from the Far Eastern Seas (Sea of Japan, SOJ, and Okhotsk Sea, OS) during the period of 1998–2021. The ^137^Cs activity levels showed exponential changes during the observed period. The decay-corrected change rates of ^137^Cs in deep waters of SOJ exhibited a slow increase, while ^137^Cs levels in seawater and sediment in OS decreased gradually. This reflects a topographical difference, as SOJ is a semi-closed sea, whereas OS receives continuously inflow of subarctic waters. It was confirmed that ^137^Cs released after the Fukushima Dai-ichi Nuclear Power Plant accident was rapidly transported into the deep waters of the SOJ. To elucidate the transfer processes of ^137^Cs from seawater to sediment, we discussed the temporal changes of the partition coefficients (K_d_) of ^137^Cs between the overlying water and the surface sediment. In shallow areas (< 1500 m water depth), K_d_ values were almost constant within the sampling periods, although the temporal changes in the K_d_ values occurred in deeper waters (> 2500 m depth). The K_d_ values increased with increasing depth, which may reflect a pressure effect as a possible mechanism. These findings suggest that chemical processes may be important factors controlling the transport of ^137^Cs between seawater and sediment, although more complicated phenomena occurred in deep waters and sediments of the SOJ (> 3000 m depth).

## Introduction

As a result of global fallout due to large-scale atmospheric nuclear testing in the early 1960s^1,2^, seabed sediment in the world ocean has been contaminated by anthropogenic radionuclides, typically by ^137^Cs, ^90^Sr, plutonium isotopes, and ^241^Am, which have been detected in coastal and deep ocean sediments^3–6^. Although there was a low contribution of radioactive deposition in the Antarctica from atmospheric nuclear tests, ^137^Cs was detected in Antarctica surface sediments, at the Italian station and in the Ross Sea, whose activities were 0.14–1.79 and < 0.1–0.96 Bq kg^−1^, respectively^7,8^, which were the same order of magnitude as those in deep ocean sediments of the Pacific Ocean. In contrast to anthropogenic radionuclides in coastal sediments, studies of anthropogenic radionuclides in deep ocean sediments around the world were limited. Most studies have focused on hot spots, such as radioactive waste dumping sites (the Sea of Japan and Arctic Seas)^9–15^, planning areas for dumping (western North Pacific and others), near nuclear weapons testing sites (Pacific Proving Ground and Mururoa)^16,17^ and outlet areas of radioactive discharges from nuclear fuel reprocessing plants^18–20^.

Only limited knowledge is available on the transport processes of ^137^Cs from the water column to sediment in the deep ocean, although possible mechanisms have been considered including partitioning between overlying water and surface sediment, direct input of sinking particles, and deep-ward transport of resuspended shallow sediment particles^20^. To elucidate the behaviours of ^137^Cs in sediment in deep oceans, it is important to clarify the temporal changes of ^137^Cs in sediments. Monitoring of radionuclides at a candidate site for an ocean dumping of radioactive wastes gave us the opportunity to know the long-term behaviour of ^137^Cs and ^90^Sr activity concentrations in deep ocean sediments for 13 years^20^.

The Hydrographic Department of the Japan Coastal Guard (JCG) has conducted measurements of ^137^Cs in the upper 2 cm of the sediment at around 30° N and 147° E during the period from 1979 to 1992. The reported massic activities of ^137^Cs were in the range of 0.63–1.46 Bq kg^−1^ dw (dry weight). There was no clear trend in ^137^Cs activity changes during these 13 years. On the other hand, temporal changes of ^137^Cs in coastal sediment around Japan (1984–2010) before the Fukushima Dai-ichi Nuclear Power Plant (FDNPP) accident were examined, in which ^137^Cs massic activities in coastal sediments decreased with effective half-lives of 10.3–55.1 years^21^. However, no information is available on the temporal variability of ^137^Cs in SOJ and OS sediments.

The Far Eastern Seas, including SOJ and OS, have been contaminated by Russian radioactive waste disposal practices (1966–1993)^22^, as did the dumping and loss of nuclear batteries, thus contributing to global fallout, derived from atmospheric nuclear testing. To assess the effect of Russian radioactive waste dumping, measurements of anthropogenic radionuclides in sediment off Vladivostok were already conducted^13,23^. ^137^Cs massic activities in surface sediments (0–3 cm) off Vladivostok (SOJ) in 1993 were in the range of < 0.4 to 7.2 Bq kg^−1^ dw. The ^137^Cs levels in surface sediments of the Yamato Ridge, the Korea Plateau, and the Ulleung Basin (1000–2000 m water depth) in 1993 ranged from 8.3 to 17.7 Bq kg^−1^ dw. In the western Japan Basin (3300–3400 m depth), higher ^137^Cs levels in surface sediments (0–0.5 cm) were measured, 18–25 Bq kg^−1^ dw^23^, while a lower ^137^Cs level (2.6 Bq kg^−1^ dw at 2931 m water depth was measured in 1984^3^ and 0.4–9.1 Bq kg^−1^ dw in 1998–2002^24^ was observed in sediments from the eastern JB. The vertical distributions of the ^137^Cs levels in SOJ sediment cores showed surface maxima with decreasing levels with sediment depth, which coincided with those of open ocean sediment cores, for example, in the North Pacific^3,5,23^.

According to the Japanese monitoring plan, JCG^25^ has been measuring ^90^Sr, ^137^Cs and ^239,240^Pu levels in seawater and sediment of the SOJ and OS within the Japanese Exclusive Economic Zone (EEZ) since 1993. These long-time series data on anthropogenic radionuclides in seawater and sediment have been useful not only for subsequent evaluation of the impact of the FDNPP accident on the ocean but also for studying oceanographic processes in the SOJ and OS.

The SOJ is a unique marginal sea in the western North Pacific, representing a deep ocean basin (maximum depth of 3700 m) with homogeneous cold water and intrusion of warm saline water (the Tsushima Warm Current as a blanch of the Kuroshio Current) and cold low-saline water (Liman Current)^26,27^. The OS, with a maximum depth of 3372 m, is another marginal sea in the western North Pacific, where freshwater flows from the Amur River, causing ice floe in winter. A blanch of water mass of the SOJ flows to the OS via the Soya Strait. A detail of oceanographic background of SOJ and OS is described in the Supplementary document.

To better understand the temporal changes of ^137^Cs in sediments and a time revolution of ^137^Cs in overlying waters is therefore an important issue. Temporal changes in the ^137^Cs activity concentrations in surface water (0–10 m) of SOJ and OS have been studied not only to clarify the fate of global fallout ^137^Cs, but also to evaluate the effects of the Fukushima-derived ^137^Cs^28–33^. On the other hand, there was limited information on ^137^Cs in deep waters (deeper than 1000 m) of the SOJ. Miyao et al.^34^ analysed time-series ^137^Cs data in deep waters of the SOJ during the period 1976–1996. The results revealed that the ^137^Cs activity concentrations in seawater in layers below 1500 m depth increased during the period 1985–1995, which implied that significant amounts of ^137^Cs-rich surface water conveyed into the deep layers after 1985. This finding suggests that it is important to clarify the temporal change of ^137^Cs in deep waters after 1996.

**Figure 1:**
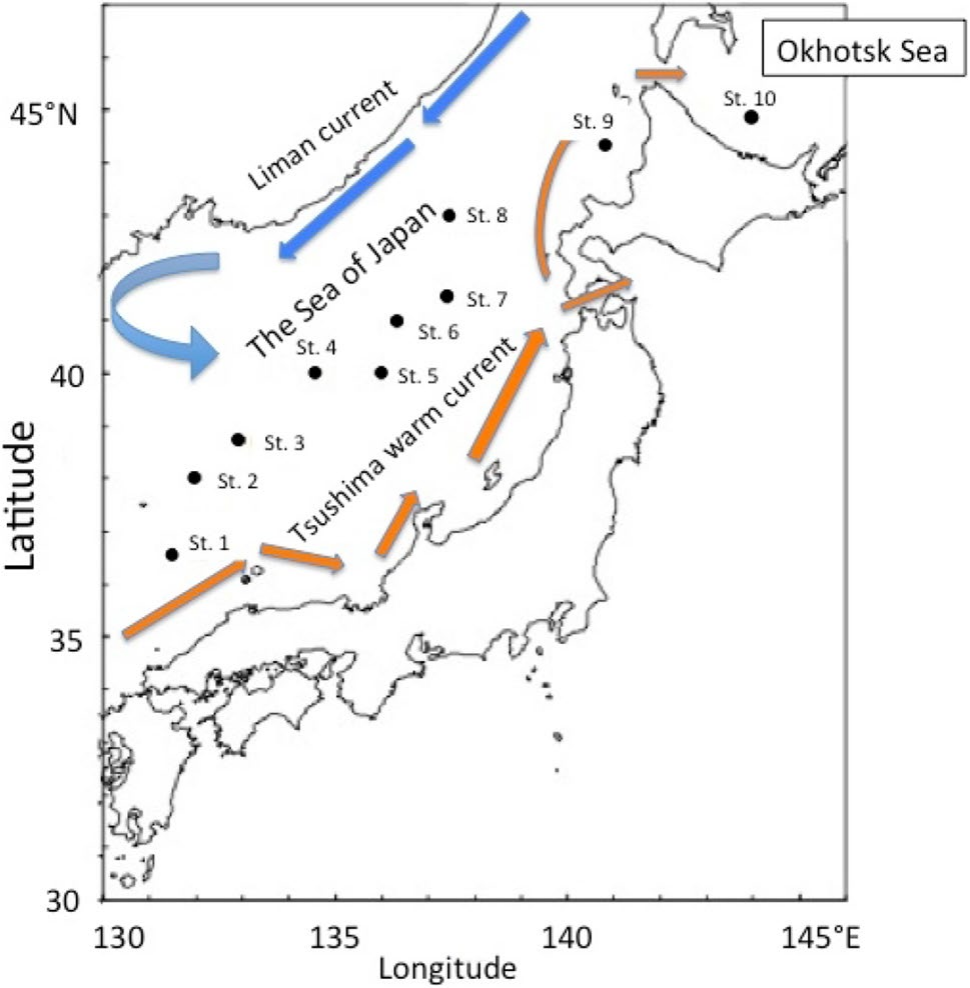
Schematic map and sampling points of the Sea of Japan and Okhotsk Sea.

In this paper, we describe the temporal changes in ^137^Cs activity concentrations in overlying waters and sediments in the SOJ and OS. We also discuss the chemical process that controls the behaviour of ^137^Cs in deep-ocean sediments. The main water sampling locations in SOJ and OS used in this work are shown in Fig. 1. Data on sampling locations, sampling results and overlying water depths of sediments are shown in Supplementary Tables S1, S2A and S2B, respectively.

## Results and discussion

### ^137^Cs in overlying waters

The results of ^137^Cs activity concentrations in the overlying waters are summarized in Table S3. The overlying water in this paper is defined as a water sample collected at a closest layer to the bottom, which is classified as the Shallow Overlying Water (SOW) (100–300 m depth), the Mid-depth Overlying Water (MDOW) (1000–2000 m depth), and the Deep Overlying Water (DOW) (deeper than about 2800 m). The MDOW and DOW correspond to the Japan Sea Proper Water (JSPW) and the Bottom Water (BW), respectively, whose oceanographic definitions are cold homogeneous water in salinity (34.04–34.10), low temperature (0.2–0.5 °C), and higher concentrations of dissolved oxygen (220–250 µmol kg^−1^), and homogeneous water below the benthic front in the JB, respectively (described in detail in the Supplementary docs). The ^137^Cs activity concentrations were high in SOW and low in DOW, which was consistent with previous results^35,36^. The variability ranges of ^137^Cs in the overlying waters were low in SOW of the SOJ (St. 9: 1.5–2.3 Bq m^−3^), and slightly higher in SOW of the OS (St. 10: 0.71–2.31 Bq m^−3^) and in MDOW (St. 1; 0.25–0.61 Bq m^−3^, St. 2: 0.34–0.73 Bq m^−3^, St. 4: 0.59–1.15 Bq m^−3^, St. 5: 0.44–1.17 Bq m^−3^) and in DOW (Tsushima Basin/Ulleung Basin: TB/UB) (St. 3: 0.23–0.54 Bq m^−3^), while they were relatively high in DOW (Japan Basin: JB) (St. 6: 0.11–0.6 Bq m^−3^, St. 7: 0.13–0.51 Bq m^−3^, St. 8: 0.14–0.82 Bq m^−3^).

**Figure 2:**
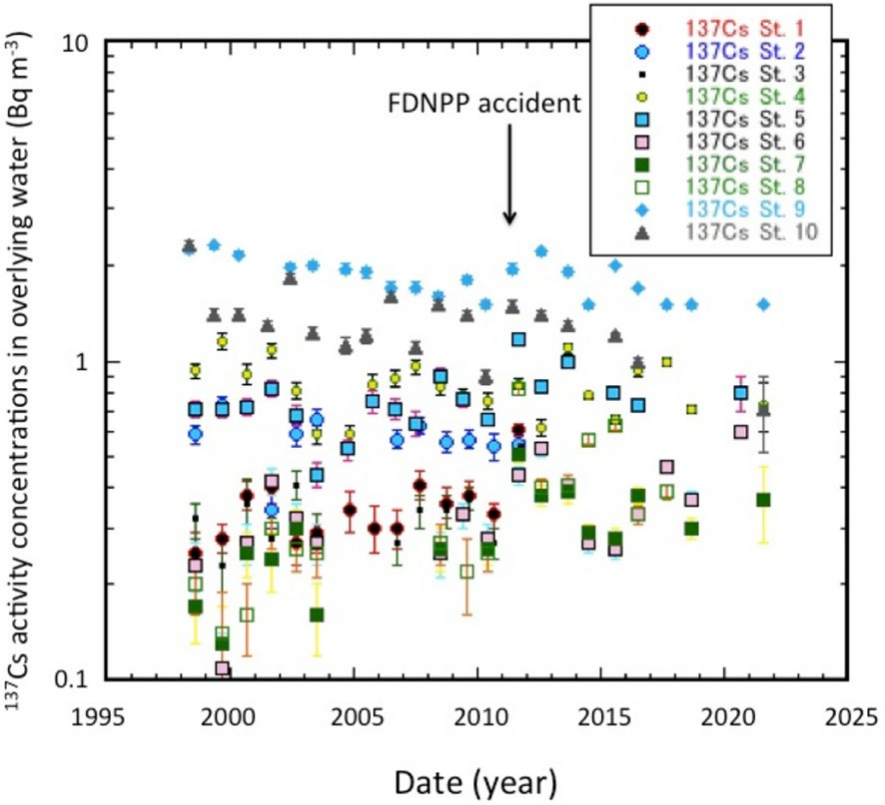
Temporal changes of ^137^Cs activity concentrations in overlying waters of the SOJ and OS.

We examine in detail the temporal changes of the ^137^Cs activity concentrations in the overlying waters in SOJ and OS (Fig. 2). After the FDNPP accident, the northern SOJ at its initial stage, the Tsushima Warm Current, and the southern OS waters were affected by the fallout and transport of FDNPP-derived ^137^Cs^30–33^^,^^37^. The high ^137^Cs activities in the overlying water compared to those in previous three years (2008–2010) occurred in SOJ in June 2011 (St. 9) and August–September 2011 (Sts. 1, 3, 5, 6, 7 and 8). On the other hand, increased ^137^Cs activity was not observed in St. 2 in September 2011. The FDNPP accident fallout showed during the period of March–May 2011 a geographic distribution with high levels in the south eastern SOJ, and low levels in the western SOJ^20^. The enhanced ^137^Cs levels in the overlying water also showed a geographic distribution (Fig. S2), in which low FDNPP-derived ^137^Cs levels occurred in TB/UB of the western SOJ, and the highest occurred in JB (St. 8) of the north eastern SOJ. This finding suggests that sinking particles, including FDNPP-derived ^137^Cs, also contributed to the dissolved ^137^Cs in deep waters. The 2011 signal of the FDNPP-derived ^137^Cs at St. 9 was relatively weak, however, it was consistent with vertical profiles of ^134^Cs in the north eastern SOJ^38^.

Although the temporal variation of the ^137^Cs activities in OS (St.10) exhibited a decreasing trend, they were divided into two groups according to the temperature of OS (Fig. S1A): the higher temperature (0.43–3.21 °C) group, affected by SOJ water, showed higher ^137^Cs activities, while the lower temperature (− 0.32 to − 1.54 °C) group, reflecting intermediate OS water, showed lower ^137^Cs activities. A contribution of FDNPP-derived ^137^Cs to the ^137^Cs activity in SOW of the SOJ (St. 9) after March 2011 was consistent with the previous result^33^.

The sediment trap experiment revealed that FDNPP-derived ^134^Cs was detected in sinking particles moored at 1100 m depth in the JB, while there was no ^134^Cs signal in sinking particles at 3500 m depth^39^. This finding suggests that FDNPP-derived ^137^Cs could affect the levels of dissolved ^137^Cs in deep water (> 1100 m depth), as documented in high sporadic ^137^Cs levels found in the overlying waters of Sts. 6, 7 and 8 in the JB in the SOJ. Therefore, we can state that the FDNPP-derived ^137^Cs was rapidly transported into the DOW of the SOJ.

**Figure 3:**
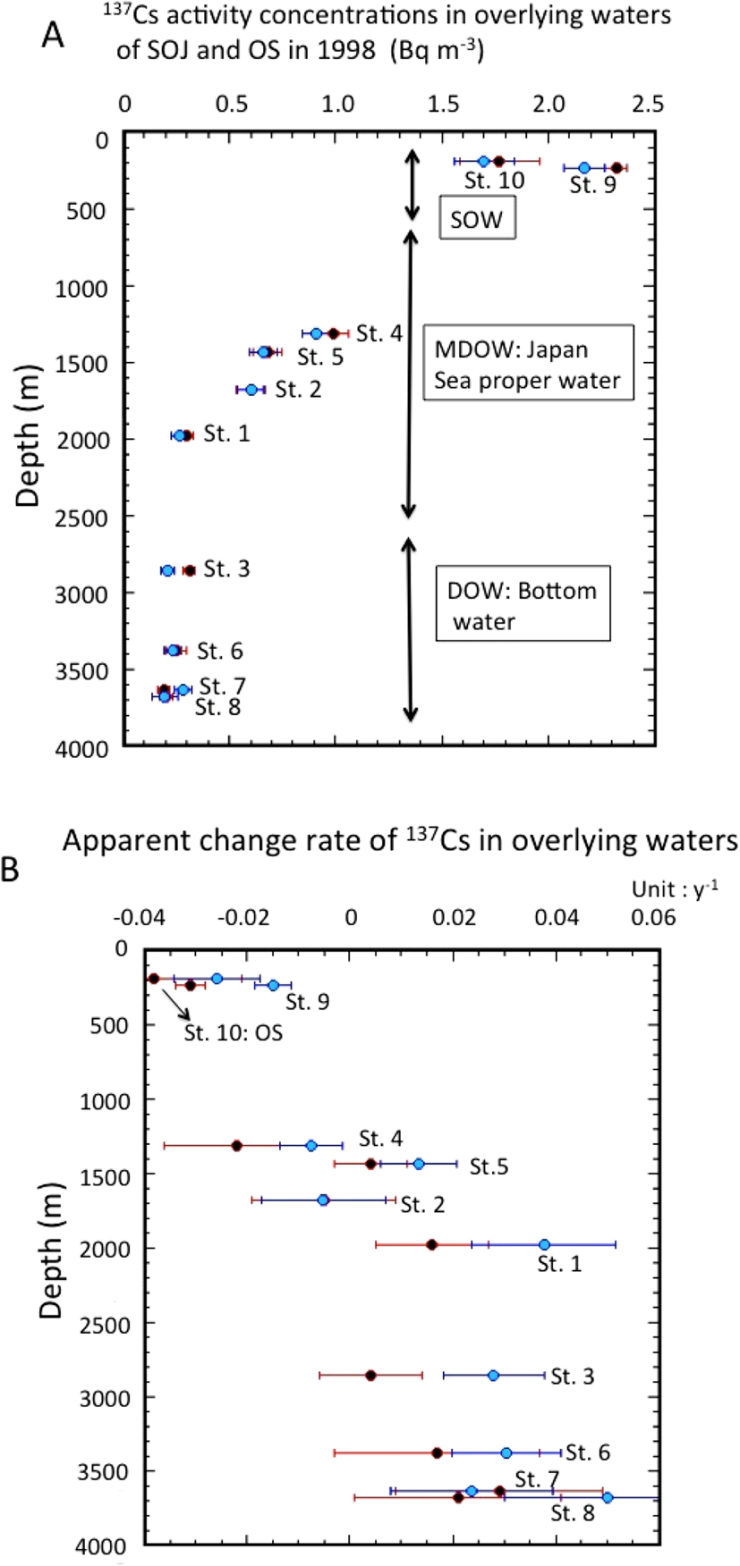
(**A**) Vertical profiles of ^137^Cs activity concentrations in overlying waters of the SOJ and OS in January 1998. Pre-Fukushima era (1998–2010): red closed circles, full data use: pale blue closed circles. (**B**) Apparent change rates of ^137^Cs in overlying waters of the SOJ and OS. Pre-Fukushima era (1998–2010): red closed circles, full data use: pale blue closed circles.

Assuming that the ^137^Cs activity concentrations in the overlying waters changed exponentially, the apparent change rates (ACR: *k*_*aw*_) were calculated in each overlying waters as follows; *C*_*137Cs,OW*_ = *C*_*137Cs,OW,o*_* exp{k*_*aw*_*(t*–*1998)}*, where *C*_*137Cs,OW*_ and *C*_*137Cs,OW,o*_ were the observed ^137^Cs activity concentrations in the overlying waters and ^137^Cs was the activity concentrations on January 1, 1998, respectively. Since there was the possibility of the influence of the fallout from the Fukushima accident, we divided the time scale into two periods: the pre-Fukushima era (1998–2010) and the full sampling period. The values of *C*_*137Cs,OW,o*_ and ACR for the pre-Fukushima era and the full sampling period are presented in Table S4. For the pre-Fukushima era, the *C*_*137Cs,OW,o*_ values in the SOJ, ranging from 0.19 ± 0.03 to 2.32 ± 0.05 Bq m^−3^, decreased from the shallow layer to approximately 2000 m depth, and showed a constant level of 0.25 Bq m^−3^ below 2000 m depth in 1998 (Fig. 3A), which is consistent with the phenomenon that ^137^Cs activity concentrations in seawaters from surface to 2000 m depth are controlled by eddy diffusion and homogeneous water mass (JSPW and BW) that exists in the SOJ waters below 2000 m depth^26^. The ACRs of ^137^Cs in the overlying waters of SOJ and OS ranged from − 0.031 ± 0.03 to 0.029 ± 0.02 year^−1^, and − 0.038 ± 0.017 year^−1^, respectively. The relationship between the ACR and depth is shown in Fig. 3B. The ^137^Cs activity concentrations in SOW (St. 9 and St. 10) exhibited slow decreasing trends during the period 1998–2010, whereas they increased with slow rates in deep waters below about 2000 m depth of the SOJ; the ACRs, ranged from 0.016 to 0.029 year^−1^, and were almost constant. This finding revealed that the ^137^Cs activity concentrations in SOJ deep waters have continuously increased since 1985. The ACRs in middle layers around 1500 m depth showed positive and negative values. The physical (decay-corrected) change rates (PCR: *k*_*pw*_), which reflect the physical oceanic processes of ^137^Cs, are calculated as follows: k_pw_ = k_aw_ + λ(^137^Cs), where λ(^137^Cs) is the radioactive decay constant. The PCRs in SOJ ranged from − 0.008 to 0.044 year^−1^, while the PCR in OS was − 0.015 year^−1^. These findings suggest that SOJ, especially JSPW, accumulates ^137^Cs by vertical mixing due to deep water formation and warm anticyclonic eddy activities^36,40^.

We also examined the temporal changes of ^137^Cs by using the complete data set to evaluate the effects of Fukushima-derived ^137^Cs (Table S4). The *C*_*137Cs,OW,o*_ values in the SOJ, ranging from 0.20 ± 0.04 to 2.17 ± 0.09 Bq m^−3^, decreased from the shallow layer to approximately 2000 m depth, and showed a constant level of 0.24 Bq m^−3^ below 2000 m depth in 1998 (Fig. 3A), which were similar to those in the pre-Fukushima era. The ACR(L)s of ^137^Cs in the overlying waters of SOJ and OS ranged from − 0.015 ± 0.004 to 0.05 ± 0.02 year^−1^, and − 0.026 ± 0.008 year^−1^, respectively. The relationship between the ACR and depth is shown in Fig. 3B. We compared between the ACR of ^137^Cs (the pre-Fukushima era: 1998–2000) and the ACR(f) (full data of 1998–2022). In the SOW, the increase rates of ACR(f)s from ACRs were 0.016 ± 0.005 year^−1^ in the SOJ (St. 9) and 0.012 ± 0.019 year^−1^ in the SO (St. 10). This finding reveals that FDNPP-derived ^137^Cs affected SOW in the SOJ due to its inflow through the Tsushima Warm Current^28,31^, while in the OS it was diluted by water with low ^137^Cs levels found there. In the MDOW, the increase rates of ACR(f)s from ACRs were 0.022 ± 0.018 year^−1^ at St. 1, 0.0 ± 0.018 year^−1^ at St. 2, 0.014 ± 0.015 year^−1^ at St. 4, and 0.009 ± 0.010 year^−1^ at St. 5. In the DOW, the increase rates of ACR(f)s from ACRs were 0.024 ± 0.014 year^−1^ at St. 3, 0.013 ± 0.023 year^−1^ at St. 6, − 0.005 ± 0.026 year^−1^ at St. 7, and 0.029 ± 0.028 year^−1^ at St. 8. ACR(f)s in the MDOW and DOW were higher than those of the pre-Fukushima era, except for Sts. 2 and 7. Therefore, the results may suggest that the effects of Fukushima fallout^30,31^ remained in SOJ deep waters. It should be noted that a part of the increase in ^137^Cs in deep water after 2011 may be due to the contribution of dissolution of FDNPP-derived ^137^Cs in sinking particles, taking into account the results of sediment trap experiments^39^.

### ^137^Cs in sediment

The results of ^137^Cs activity concentrations in surface sediment (0–2 cm) are summarized in Table S5. Unlike ^137^Cs in the overlying waters, there was no depth dependence of the ^137^Cs activities in the sediments; the lowest value occurred at TB/UB St. 3 (0.01 Bq kg^−1^), while the highest value was observed in the JB St. 6 (6.0 Bq kg^−1^). The ^137^Cs variability ranges of sediments at shallow bottom depth (St. 9 of SOJ: 1.3–3.35 Bq kg^−1^, St. 10 of OS: 1.2–2.9 Bq kg^−1^) were slightly smaller than those of JSPW (St. 1: 1.0–4.8 Bq kg^−1^, St. 2: 0.91–3.23 Bq kg^−1^, St. 4: 0.5–2.8 Bq kg^−1^, St. 5: 1.1–3.1 Bq kg^−1^), while those values were markedly large in BW (St. 3: 0.01–0.54 Bq kg^−1^, St. 6: 1.1–6.0 Bq kg^−1^, St. 7: 0.18–3.7 Bq kg^−1^, St. 8: 0.02–0.35 Bq kg^−1^). The spatial and temporal variability of ^137^Cs in the sediments of the JB was greater than in the overlying waters.

**Figure 4:**
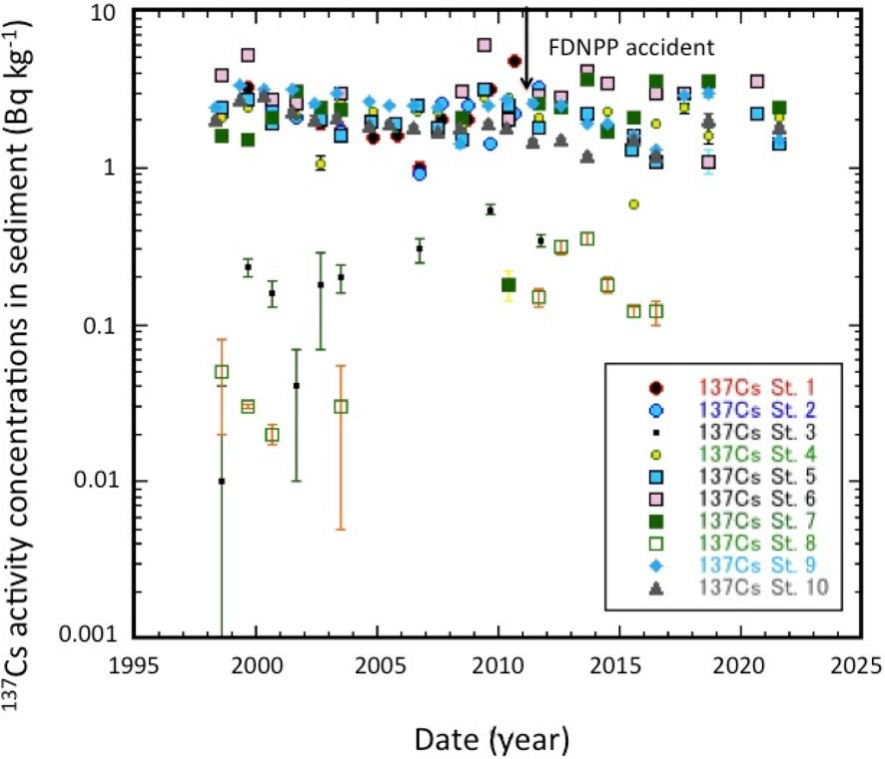
Temporal changes of ^137^Cs activity concentrations in sediments of the SOJ and OS.

**Figure 5:**
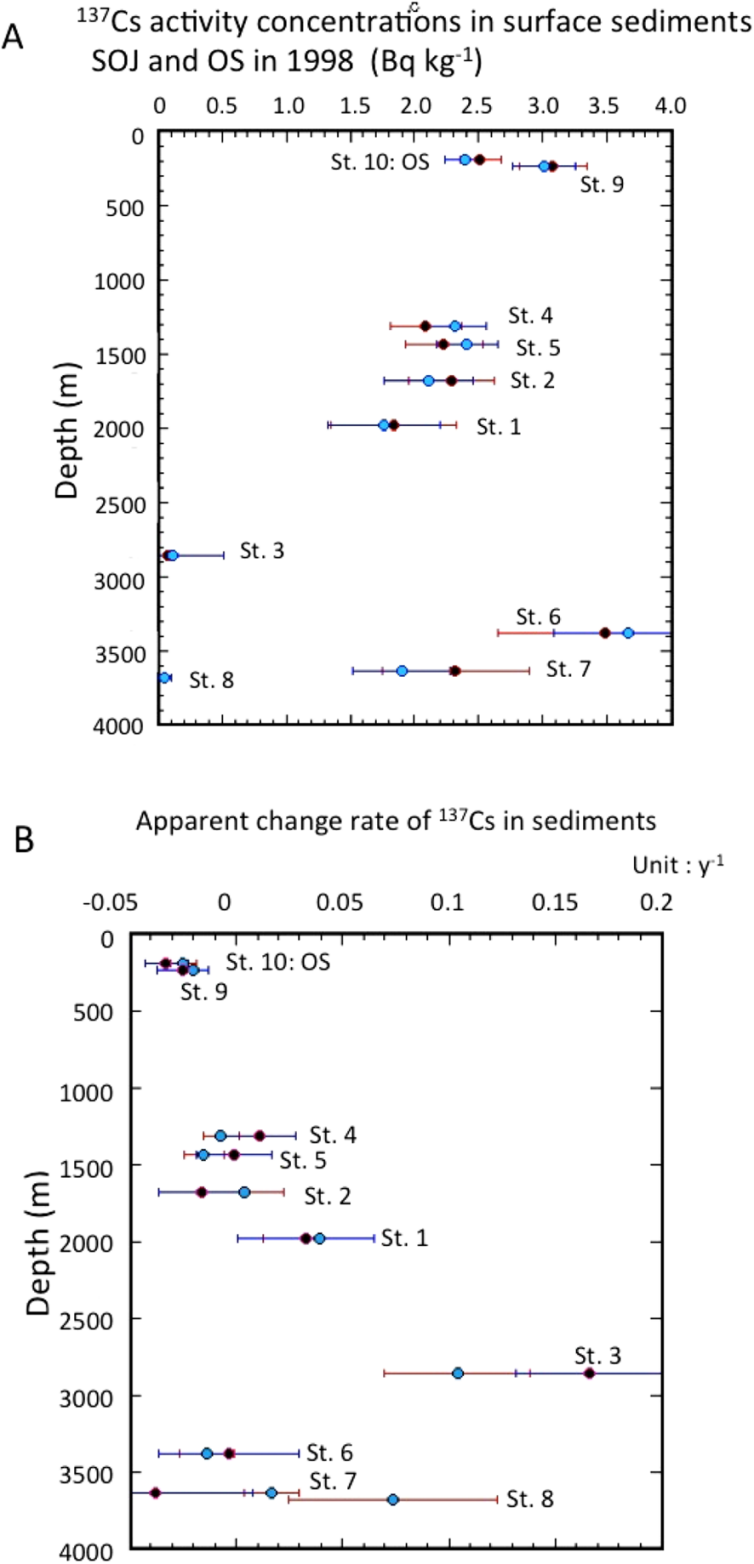
(**A**) Vertical profiles of ^137^Cs activity concentrations in sediments of the SOJ and OS. Pre-Fukushima era (1998–2010): red closed circles, full data use: pale blue closed circles. (**B**) Apparent change rates of ^137^Cs in sediments of the SOJ and OS. Pre-Fukushima era (1998–2010): red closed circles, full data use: pale blue closed circles.

To better understand the behaviour of ^137^Cs in sediments, it is important to examine the temporal changes of the ^137^Cs activities of sediments. We examine the temporal changes of ^137^Cs activities in surface sediment (0–2 cm) of the SOJ and OS during the period 1998–2021. The results are shown in Fig. 4. After the FDNPP accident, in contrast to the overlying water, higher ^137^Cs activities in sediments in August–September 2011 were observed only at two sites (Sts. 6 and 7). Assuming that the ^137^Cs activity of the sediments changed exponentially, apparent change rates (ACR: *k*_*as*_) of ^137^Cs in the sediments were calculated in as follows; *C*_*137Cs,SS*_ = *C*_*137Cs,SS,o*_* exp{k*_*as*_*(t* − *1998)}*, where *C*_*137Cs,SS*_ and *C*_*137Cs,SS,o*_ were observed ^137^Cs activities in sediments (dry base) and ^137^Cs activity on January 1, 1988, respectively. Since there was a possibility of influence of Fukushima fallout, we divided the time scale in two periods: pre-Fukushima era (1998–2010), and the full sampling period. The calculated values for the pre-Fukushima era and the full sampling period are presented in Table S6. For the pre-Fukushima era, the *C*_*137Cs,SS,o*_ values were in the range of 1.84 ± 0.49 to 3.48 ± 0.83 Bq kg^−1^, in which the *C*_*137Cs,SS,o*_ values in the SOJ gradually decreased from the 256 m layer to 2000 m depth, and showed great variability below 2000 m depth (Fig. 5A). The spatial distribution of *C*_*137Cs,SS,o*_ in the JB was different from that of the overlying water ^137^Cs. The ACRs of ^137^Cs in surface sediments of SOJ ranged from − 0.038 ± 0.046 to 0.033 ± 0.032 year^−1^, and the ACR in OS was − 0.033 ± 0.010 year^−1^. The relationship between the ACR and depth is shown in Fig. 5B. The ^137^Cs levels in SOJ sediments (except St. 1 and St. 4) exhibited slow decreasing trends during the period 1998–2010 (ACR: − 0.033 to − 0.001), while in St. 1 and St. 4, they increased with slow rates; the ACRs, ranged from 0.011–0.033 year^−1^. Compared to ACRs in the overlying waters, the depth dependence of ACRs in sediments was not clear, although there was large variability of ACRs below 2000 m water depth. The physical change rates (PCR) of ^137^Cs in sediments were calculated, as did those of overlying waters. The PCRs in SOJ exhibited positive values (0.007–0.066 year^−1^), except for Sts. 7 and 9, while those in OS showed a negative value (− 0.01 year^−1^). This finding suggests that the global fallout-derived ^137^Cs in SOJ has gradually transferred from the water column to the sediment, following its accumulation in the water column. Kusakabe and Takata^21^ reported temporal changes in ^137^Cs activity concentrations in coastal waters and sediments around Japan during the period 1984–2010. The observed effective half-lives of ^137^Cs in coastal sediments were slightly longer than in seawater samples (23.6 years and 17.3 years, respectively, corresponding to − 0.029 and − 0.04 year^−1^ of the apparent change rates, respectively). However, it is a further issue why the large spatiotemporal variability of ^137^Cs occurred in surface sediments of deep SOJ (> 2000 m water depth: i.e., in lower JSPW and BW), regardless of the finding that ^137^Cs in the deep-water column (> 2000 m water depth) was relatively homogeneous. An explanation could be that deep water sediments may be perturbed by ocean floor currents, e.g., by tidal currents^41^.

We also examined the temporal changes of ^137^Cs by using the full data set to evaluate the effects of FDNPP-derived ^137^Cs. The *C*_*137Cs,SS,o*_ values in SOJ, ranging from 1.74 ± 0.44 to 3.67 ± 0.58 Bq kg^−1^, were similar to those in the pre-Fukushima era (Fig. 5A). The ACRs of ^137^Cs in the sediments of SOJ and OS ranged from − 0.02 ± 0.007 to 0.039 ± 0.026 year^−1^, and − 0.025 ± 0.006 year^−1^, respectively. The relationship between the ACR and depth is shown in Fig. 5B. Compared with ACRs of ^137^Cs during the pre-Fukushima era, there were no clear enhanced ACRs for full data use (except for St. 9 and 10). Therefore, we cannot find that a distinct signal of FDNPP-derived ^137^Cs may appear in SOJ sediments (except St. 6 and 7) due to the very sparse data density and the slow sorption–desorption reaction rates of ^137^Cs between overlying water and sediment^21^.

**Figure 6:**
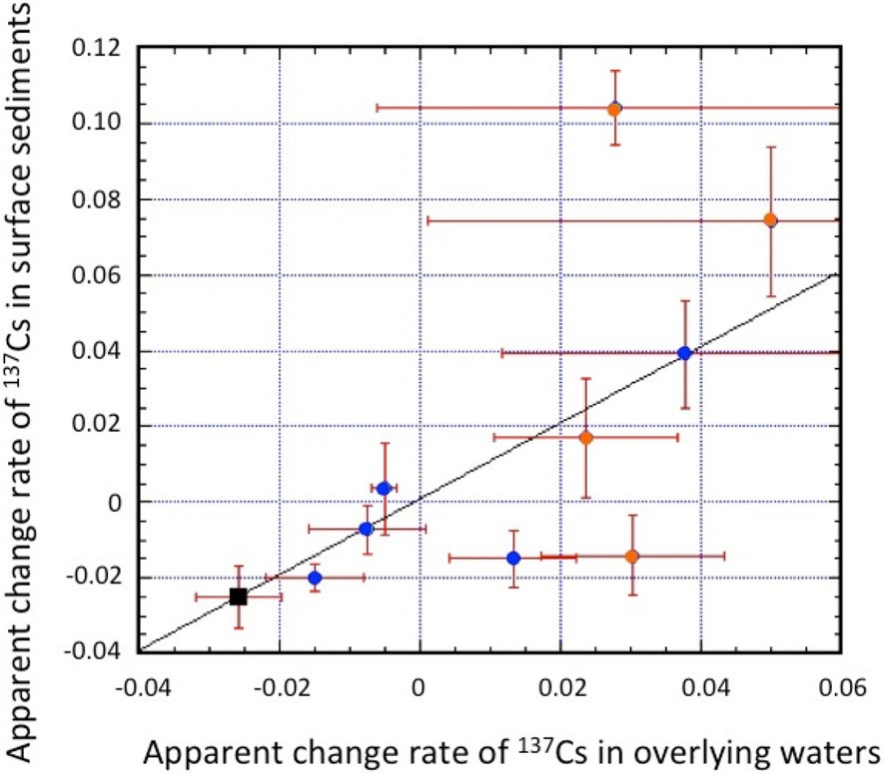
Relationship between apparent change rates of ^137^Cs in overlying waters and sediments. Red closed circles: Bottom Water (> 2800 m depth) in the SOJ, blue closed circles: shallow water and JSPW (100–2000 m depth).

The relationship between the ACRs of ^137^Cs in surface sediments and in overlying water is presented in Fig. 6. The ACRs of ^137^Cs in surface sediments of SOJ and OS above 2000 m water depth were similar to those in overlying waters, while those in sediments below 2000 m water depth showed great variability, in which the increases of ^137^Cs in surface sediments at St. 3 were larger than those in the corresponding overlying waters.

### Partition coefficients of ^137^Cs

The partition coefficient has been used as an effective tool for understanding the exchange processes of chemical substances between the liquid and solid phases. The partition coefficients of anthropogenic radionuclides between water and sediment/soil phases have been applied in radiological assessment models in aquatic systems, including freshwater and saline water systems^42,43^. The determination of the partition coefficients of radionuclides for sediments in deep oceans by experiment is a difficult task due to pressure effects. Therefore, in this paper we introduce a practical partition coefficient of ^137^Cs between deep overlying water and sediment, defined as follows:

1 $$ K_{d} = C_{137Cs, \, ss} /C_{137Cs,ow} $$

where C_137Cs, ss_ and C_137Cs,ow_ are the ^137^Cs activities in surface sediment (dry weight, 0–2 cm depth) and in overlying water (bottom-up to 150 m), respectively. The temporal variability of distances from the sea floor to the lowest observed layer was insensitive to the ^137^Cs activities in the overlying water (see Suppl./Distances and Table S9). The calculated practical partition coefficients of ^137^Cs using Eq. (1) are summarized in Table S7. The K_d_ values in shallow sediments (0.76–2.1) × 10^3^ L kg^−1^: St. 9 and 10), were of the same order of magnitude as those in coastal waters of the SOJ^21^. In deep sediments (> 3500 m water depth), high K_d_ values (> 10^4^ L kg^−1^) were observed.

**Figure 7:**
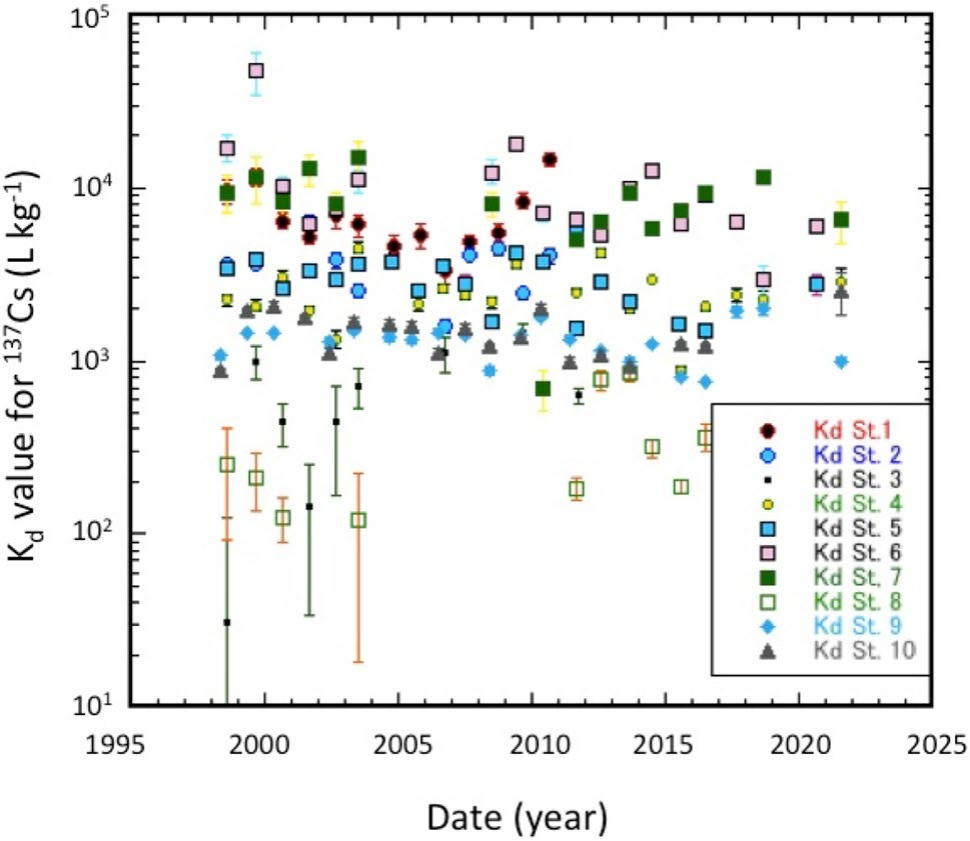
Temporal changes of partition coefficients (K_d_) of ^137^Cs between overlying waters and sediments of the SOJ and OS.

**Figure 8:**
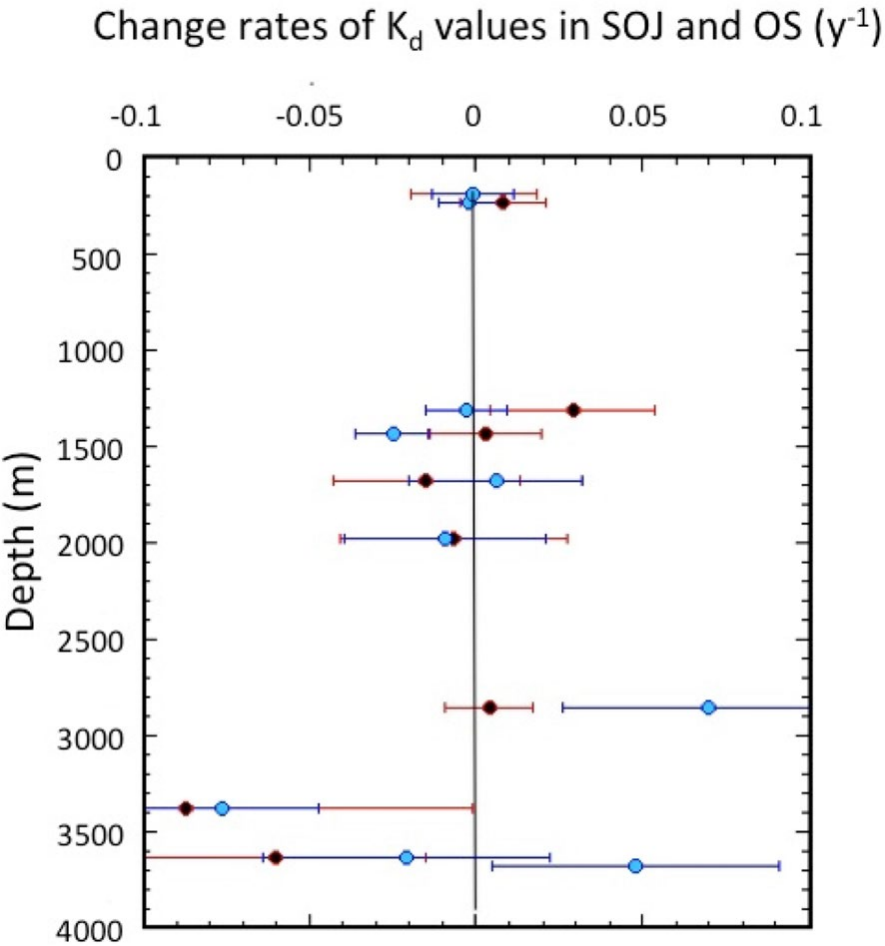
Vertical variability of change rates of partition coefficients of ^137^Cs between overlying waters and sediments of the SOJ and OS. Pre-Fukushima era (1998–2010): red closed circles, full data use: pale blue closed circles.

In addition, we examine temporal changes of K_d_ values in SOJ and OS (Fig. 7). The apparent change rates of K_d_ were calculated by assuming an exponential change process, as did ^137^Cs in the overlying waters and sediments. We divided the time scale into two periods: the pre-Fukushima era (1998–2010), and the full sampling period. The results are summarized in Table S8. For the pre-Fukushima era, the partition coefficients in 1998 (K_d,o_) in the SOJ were from (1.30 ± 0.13) × 10^3^ to (23.5 ± 9.5) × 10^3^ L kg^−1^, and (1.54 ± 0.22) × 10^3^ L kg^−1^ in OS. The apparent change rates (ACR_Kd_) of the K_d_ in the SOJ ranged from − 0.0067 ± 0.034 to 0.029 ± 0.025 year^−1^, while that in OS was − 0.0007 ± 0.019 year^−1^. Figure 8 shows a relationship between ACR_Kd_ and the depth. The ACR_Kd_ values at depths shallower than 3000 m were nearly zero (except of St. 4), taking into account uncertainties, which means that the partitioning of ^137^Cs between the overlying water and the surface sediment was apparently in equilibrium. On the other hand, the ACR_Kd_ values in deep waters (St. 6 and St. 7) were in disequilibrium regarding the partitioning of ^137^Cs between the overlying water and the sediments, whose causes are unknown.

No significant temporal and spatial changes in the mineralogical properties of sediments are expected to occur at deep ocean depths. Although the morphology of sediments in the SOJ changed during 2014–2021 from mud to clay/ooze (see Suppl./Sediment and Table S10), this change should not significantly affect ^137^Cs levels in deep water sediments, which are mainly sensitive to grain size and organic matter content of sediments^20^.

As did overlying waters and sediments, we examined temporal changes of K_d_ by using the full data set. The partition coefficients in 1998 (K_d,o_) in the SOJ were from (1.36 ± 0.15) × 10^3^ to (22.1 ± 5.3) × 10^3^ L kg^−1^, and (1.45 ± 0.2) × 10^3^ L kg^−1^ in OS. The apparent change rates (ACR_Kd_) of K_d_ in the SOJ ranged from − 0.067 ± 0.029 to 0.006 ± 0.026 year^−1^, while in OS it was − 0.001 ± 0.012 year^−1^. Figure 8 shows a relationship between ACR_Kd_ and depth, which was similar to those for the pre-Fukushima era up to 2000 m water depth. In deep layers greater than 2500 m water depth, ACR_Kd_ values increased after the Fukushima accident. We cannot decide whether this change causes the effects of Fukushima accident or not, because there was large variability of the K_d_ in the deep sediment (> 2000 m water depth).

### ***Factors controlling K***_***d***_

The partition coefficient of ^137^Cs between water and sediment is closely related to chemical processes^18^:

2 $$^{137} Cs^{ + } + \, SSM \, =^{137} CsSS \, + \, M^{ + } $$

The equilibrium constant is defined as follows:

3 $$ \begin{aligned} K_{137CsSS} & = {{\left[ {^{137} CsSS} \right]\left[ {M^{ + } } \right]} \mathord{\left/ {\vphantom {{\left[ {^{137} CsSS} \right]\left[ {M^{ + } } \right]} {\left( {\left[ {^{137} Cs} \right]\left[ {SSM} \right]} \right)}}} \right. \kern-0pt} {\left( {\left[ {^{137} Cs} \right]\left[ {SSM} \right]} \right)}} \\ & = {{K_{d} \left[ {M^{ + } } \right]} \mathord{\left/ {\vphantom {{K_{d} \left[ {M^{ + } } \right]} {\left[ {SSM} \right]}}} \right. \kern-0pt} {\left[ {SSM} \right]}} \\ \end{aligned} $$

**Figure 9:**
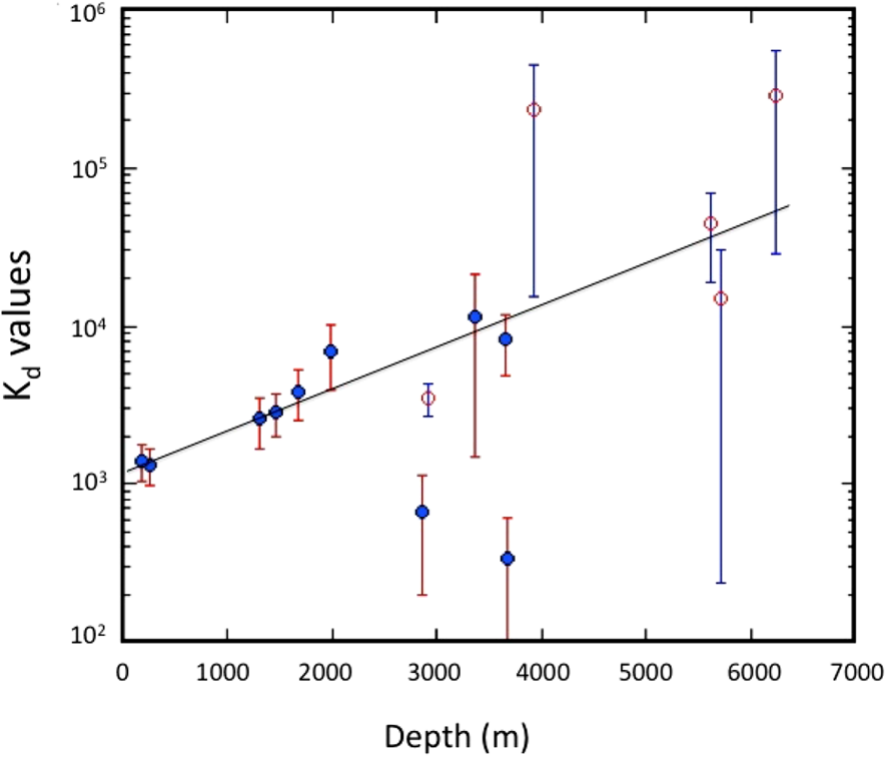
Depth dependency of partition coefficients of ^137^Cs between overlying waters and sediments of the SOJ and OS. Red open circle: western North Pacific (data from Nagaya and Nakamura^3^).

Under seawater conditions, *M*^+^ (alkali metal ions) and *SSM* are constant because of the conservative elements. According to the thermodynamics of chemical equilibrium^44^, the equilibrium constant is expressed as a function of pressure, volume, and temperature. The logarithmic equilibrium constant is a linear function of pressure when the volume is changing (*ΔV*_*o*_), and the absolute temperature (*T*) is constant.

4 $$ log\left( {K_{P2} /K_{P1} } \right) = - \Delta{V_{o}} (P2 - P1)/2.3RT, $$

where *R* is the gas constant. If the volume change accompanied by a chemical reaction is negative, a positive linear relationship is expected between the log (*K*_*P2*_*/K*_*P1*_) and the pressure change (*P2*–*P1*). Pressure (*P*) is linearly related to depth (*z*) as follows: *P* = *Gρz*, where *G* and *ρ* is the gravity constant and the density of seawater, respectively. In SOJ, the water temperature of the overlying water was in the range of 0.2–0.4 °C except for St. 9 (Supplementary Fig. S1), while in OS it was around 0 °C. This means that the absolute temperature of the overlying water was constant within the anomaly of less than 2%. The K_d_ of ^137^Cs between seawater and sediment, plotted as a function of depth, is shown in Fig. 9, in which the K_d_ values calculated from the data observed in deep sediments of the western North Pacific^3^ were included. The results revealed that the logarithmic K_d_ value of ^137^Cs was linearly related to depth. This finding suggests that the ^137^Cs—sediment interaction is stabilized under high pressure. In this connection, the K_d_ of stable Cs for coastal sediment was 1.7 × 10^3^ (Ref.^45^), while the K_d_ of stable Cs in deep sediment was calculated to be 1.7 × 10^4^ (Ref.^46^). The sediment trap experiment^39^ revealed that ^137^Cs levels in sinking particles increased with increasing depth. These findings suggest that the pressure effect may be important in controlling the partitioning of radionuclides between water and sediments.

## Conclusions

In the deep ocean, there is generally limited information on the behaviour of ^137^Cs in the overlying waters and sediments. Long-term monitoring of marine radioactivity, including deep sediments, conducted in the Sea of Japan and Okhotsk Sea provided useful information on processes that occurred in the overlying water and sediments in the deep ocean.

Analysis of time-series data suggests that the JSPW and BW have gradually accumulated global-fallout ^137^Cs due to the semiclosed character of the Sea of Japan, whereas ^137^Cs levels in the Okhotsk Sea decreased between 1998 and 2022. Although the impact of the 2011 FDNPP accident in the deep waters of SOJ was not obvious due to the missing ^134^Cs data, relatively high ^137^Cs activity concentrations occurred just after the FDNPP accident in the overlying waters at Sts. 6, 7 and 8 in the JB of the SOJ. We can state that the FDNPP-derived ^137^Cs was rapidly transported to the DOW of the SOJ.

The ^137^Cs levels in sediments of the SOJ gradually decreased with increasing depth up to a depth of 2000 m, whereas below this depth they showed large variations. The temporal changes of the ^137^Cs levels in sediments of the SOJ (representing the first published results) showed increasing trends in deep sediments below 2000 m water depths, although large variabilities were observed. In shallower sediments than 1500 m water depth, including the OS, the ^137^Cs gradually decreased with time, whose apparent change rates were similar to those in overlying waters.

The partition coefficients of ^137^Cs between overlying water and sediment are an important parameter in understanding chemical processes related to the exchange of ^137^Cs between liquid and solid phases. In shallower sediments, the partition coefficients were almost constant for about two decades, which may lead to the hypothesis that a chemical equilibrium regarding ^137^Cs exchange or adsorption/desorption was established between the overlying water and the sediment. If the partition coefficients were controlled by chemical processes, they were influenced by hydraulic pressure (or water depth). The presented observations suggest that the logarithmic partition coefficients were linearly related to the depth.

The importance of chemical factors has been proposed to understand the behaviours of ^137^Cs between overlying water and sediment. However, there are still unknown matters influencing the behaviour of ^137^Cs in deep water sediments of the SOJ, for example why ^137^Cs levels in sediments have been varying temporally and spatially? To better understand the behaviour of ^137^Cs in the overlying waters and sediments of the SOJ, further studies are required, including new sampling missions and modelling.

## Methods

^137^Cs JCG data reported for the period of 1988–2021^25^ with one sampling per year were used in this study. The main water sampling locations in SOJ and OS used in this work are shown in Fig. 1. Data of sampling locations and overlying water depths of sediments are shown in Supplementary Table S1. Deep waters and sediments were collected on board using a 100 L water sampler and an improved Smith-Macintyre sampler, respectively. The overlying water is conventionally defined as the deepest layer in each site. Surface sediments were collected in the 2 cm top layer. ^137^Cs in seawater was concentrated with AMP after a spike of stable Cs. Seawater after purification with palatinate and ^137^Cs coprecipitation, was analysed by a low-background beta spectrometer. The sediment sample (100 g) was dried and pulverized, then passed through a sieve with a mesh of 2 mm. After the decomposition of organic matter at 470 °C, ^137^Cs was extracted in 8 M HCl solution. The ^137^Cs in solution was then concentrated, purified and measured similarly to the case of seawater.

The sampling status, and ^137^Cs activity concentration ranges in the overlying water and in the sediments are presented in Supplementary Tables S2, S3 and S4. Morphological information on sediments is presented in Suppl. Table S10.

### Supplementary Information

Supplementary Information.

## Data Availability

The datasets used during the current study available from KH (hirose45037@mail2.accsnet.ne.jp) on reasonable request.
