# Supplementary material for: Temporal changes of 137Cs concentrations in the Far Eastern Seas: partitioning of 137Cs between overlying waters and sediments
Source: Sci Rep. 2023 Dec 27;13:22963. doi: 10.1038/s41598-023-49083-4 (PMC10752905; doi:10.1038/s41598-023-49083-4)
Supplement: Supplementary file 1 — Supplementary Information. [file 41598_2023_49083_MOESM1_ESM.docx]

**Supplement documents**

**Oceanographic background and supplementary results**

The oceanographic background of the Sea of Japan (SOJ) is described in detail by Hirose and Povinec^1^. The SOJ is a unique marginal sea characterized by a semi-closed feature with maximum depth of 3742 m, connecting to the East China Sea, the North Pacific Ocean and the Okhotsk Sea through shallow straits (Tsushima, Tsugaru, Soya, and Tatarskiy (Mamiya)) with less than 150 m water depth. The warm Tsushima Current, characterized as a branch of the Kuroshio Current, inflows into the SOJ, while the Liman Current, associated with cold and low saline water from the Tatarskiy Strait is present in the northern SOJ as a cyclonic circulation^2^. The deeper water layers in the SOJ (more than a few hundred meters) are occupied by an almost homogeneous cold-water mass, characterized as Japan Sea Proper Water (JSPW), which is strongly homogeneous in salinity (34.04 - 34.10), low temperature (0.2 - 0.5 °C), and higher concentrations of dissolved oxygen (220-250 µmol kg-1)^3^. In this work, the overlying waters at stations 1 – 5 correspond to the JSPW, which is formed by deep convection offshore Vladivostok during the winter monsoon period^4^. Furthermore, bottom water (BW) below benthic front of 2000 – 2500 m depth exists in the Japan Basin, characterized as a vertically homogeneous layer with fluctuation of potential temperature < 0.001 °C^5^. In this work, the overlying waters at stations 6 - 8 correspond to BW. Other interesting features are large seasonal variations of sea surface temperature (SST) by more than 15 °C, the presence of active biological processes and of polar (or sub-polar) front between the northern cold (subpolar) and southern warm (subtropical) water masses. The polar front showed interannual changes: It migrates more widely (36°N – 41°N) in the western part of the SOJ than in the eastern part^6^. The SOJ is also one of the most eddy active seas. Anticyclonic (warm) eddies are formed in the Tsushima Current region^7-10^.

The SOJ has been affected by global warming^1^, where the SST in the central SOJ has shown high increase rates of 1.87 °C/100 y^11^, which exceeds the SST increase rates of all oceans (1.27 °C/100 y), as well as of the surface air temperature (1.30 °C/100 y) during the past about 100 years. In particular, SST in the central SOJ during the winter season (January – March) exhibits the highest rate of increase (2.54 °C/100 y) within the sea areas surrounding the Japan islands. The potential temperature of the upper JSPW (800 m depth) has also been increasing with a rate of 0.16 ± 0.09 °C/100 y during the period 1958 – 1966 (ref. 12). There are current temporal changes in the water column of the SOJ. Figure S1 shows temporal changes in temperature in subsurface water (around 200 m depth), JSPW (1300 – 2000 m depth) and BW (2600 – 3670 m depth) during the period of 1998 - 2021.

In 2000, clear signals of the decrease in water temperature appeared in the JSPW and BW (Supplement Fig. S1). Comparison of ^137^Cs activity concentrations between 1999 and 2000 exhibited an increase from 0.1 to 0.16 Bq m^-3^ (except of St. 4, 5 and 8), which may be explained by a hypothesis that deep-water masses, formed by intrusion of shallow cold-water mass including enriched ^137^Cs owing to severe winters off Vladivostok 2000 before, were transported in wide areas of the SOJ. On the other hand, deep-water formations have been reported in the 2000 - 2001 winter^13,14^. Observation of dissolved oxygen in the water column revealed that the shallow water mass, including enriched dissolved oxygen, intruded into the bottom off Vladivostok in 2000 - 2001, due to severe winters^35-39^. However, anomalous temperature decrease corresponding to the 2000 – 2001 event did not appear after 2001. These findings suggest that there is a clear time discrepancy between this signal in 2000 and deep-water formation in the winter of 2000 – 2001.

An increase of water temperatures was observed in all water masses in the SOJ, except for 2000^15^. The increase rates of water temperature were calculated based on exponential change; increase rates in JSPW were 0.28 °C/100 y (St. 1) and 0.43 °C/100 y (St. 5), and in BW 0.4 °C/100 y (St. 4), 0.25 °C/100 y (St. 6), 0.23 °C/100 y (St. 7) and 0.23 (St. 8). This finding reveals that after the 2000-2001 event, a distinct formation of JSPW and BW did not occur in the SOJ, which may reflect the effects of global warming^16^.

The Okhotsk Sea is a marginal sea in the western North Pacific Ocean, with a mean depth of 859 m and a maximum depth of 3372 m. The Okhotsk Sea is connected with the North Pacific Ocean by passes through the Kuril Island Chain; two straits are deeper than 1000 m. In winter, a significant part of the Okhotsk Sea is covered by ice floes, formed due to large amounts of fresh water from the Amur River, which is lowering the salinity of the upper water. The Okhotsk Sea is a unique sea, in which Okhotsk Sea Intermediate Water (OSIW) is formed from the Dense Shelf Water, occurring in the northwest shelf region, Western Subarctic Water originating from the North Pacific, and Soya Warm Current, which has warm and saline properties, originating from the Sea of Japan. OSIW flows from the Okhotsk Sea to the western North Pacific and contributes to Oyashio Intermediate Water^17^. Therefore, in contrast to SOJ, the Okhotsk Sea cannot accumulate contaminants, including anthropogenic radionuclides, because a significant part of Okhotsk Sea Water is continuously replaced by Western Subarctic Water with lower ^137^Cs derived from global fallout^18^, although a part of the western subarctic water was affected by the deposition of FDNPP-derived radionuclides^19^.

**References**

1. Hirose, K., Povinec, P.P. Temporal variability of plutonium in surface waters of the Sea of Japan. *J. Environ. Radioact.* **248**, 106890 (2022).
2. Martin, S., Kawase, M. The southern flux of sea ice in the Tatarskiy Strait, Japan Sea and the generation of the Liman Current. *J. Marine Res.* **56,** 141-155.(1998).
3. Sudo, H. A note on the Japan Sea Proper Water. *Prog.* *Oceanogr.* **17,** 313–336 (1986).
4. Kawamura, H., Wu, P. Formation mechanism of Japan Proper Water in the flux center off Vladivostok. *J. Geophys. Res*. **103**, 21611-21622 (1998).
5. Gamo, T., Horibe, Y. Abyssal circulation in the Japan Sea. *J. Oceanogr. Soc. Japan*. **39**, 220-230 (1983).
6. Choi, B.-J., Haidvogel, D.B., Cho, Y.-K. Interannual variation of the Poler Front in the Japan/East Sea from summertime hydrography and sea level data. *J. Mar. Sys.* **78,** 351-362 (2009).
7. Gordon, A.L. *et al.* Japan/East Sea thermocline eddies. *J. Phys. Oceanogr.* **32**, 1960-1974 (2002).
8. Hogan, P.J., Hurlburt, H.E. Why do intrathermocline eddies form in the Japan/East Sea? A modeling perspective. *Oceanogr.* **19**, 134-143 (2006).
9. Isoda, Y. Warm eddy movements in the eastern Japan Sea. *J. Oceanogr.* **50**, 1-15 (1994).
10. Jacobs, G.A., Hogan, P.J., Whitmer, K.R. Effects of eddy variability on the circulation of the Japan/East Sea. *J. Oceanogr.* **55**, 247-259 (1999).
11. JMA, Japan Meteorological Agency. Long-range change of sea surface temperature near Japan (2023). https://www.data.jma.go.jp/kaiyo/data/shindan/a_1/japan_warm/japan_warm.html
12. Minami, H., Kano, K., Ogawa, K. Long-term variations of potential temperature and dissolved oxygen of the Japan Sea Proper Water. *J. Oceanogr.* **55**, 197-205 (1999).
13. Senjyu, T. *et al.* Renewal of the bottom water after the winter 2000–2001 may spin-up the thermohaline circulation in the Japan Sea. *Geophys. Res. Lett.* **29,** 10.1029/2001GL014093 (2002).
14. Talley, L.D. *et al.* Deep convection and brine rejection in the Japan Sea. *Geophys. Res. Lett.* **30**, 1159 (2003).
15. JCG, Japan Coastal Guard. Annual reports of Radioactivity survey for 2001 – 2021 (2022) (in Japanese). <http://www1.kaiho.mlit.go.jp/KANKYO/OSEN/housha.html>.
16. Kim, K. *et al.* Warming and structural changes in the East (Japan) Sea: A clue to future changes in global oceans? *Geophys. Res Lett*. **28**, 3293-3296 (2001).
17. Itoh, M., Ohshima, K.I., Wakatsuki, M. Distribution and formation of Okhotsk Sea Intermediate Water: An analysis of isopycnal climatological data. *J. Geophys. Res*., **108**, C8, 3258 (2003).
18. Aoyama, M., K. Hirose, K. Igarashi, Y. Re-construction and updating our understanding on the global weapons tests ^137^Cs fallout. *J. Environ. Monitoring* **8**, 431-438 (2006).
19. Honda, M.C. *et al.* Dispersion of artificial caesium-134 and -137 in the western North Pacific one month after the Fukushima accident. *Geochem. J.* **46**, 1-9 (2012).

**Figure captions**


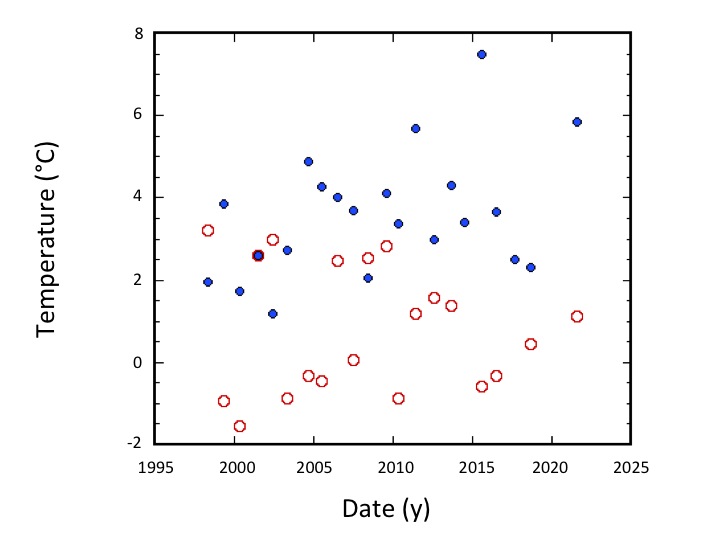


Fig. S1A. Temperature change of shallow waters (St. 9: closed circles, St. 10: open circles).


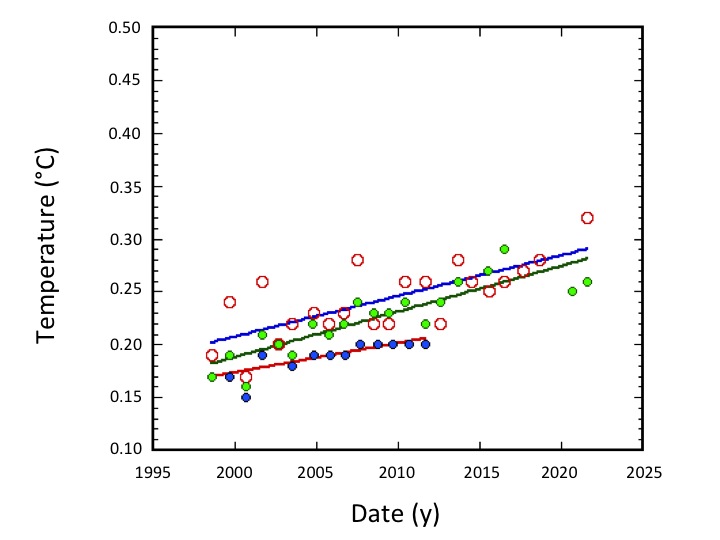


Fig. S1B. Temperature change of Japan Sea Proper Water (St.1: blue closed circles, St. 4: red open circles, St. 5: green closed circles)


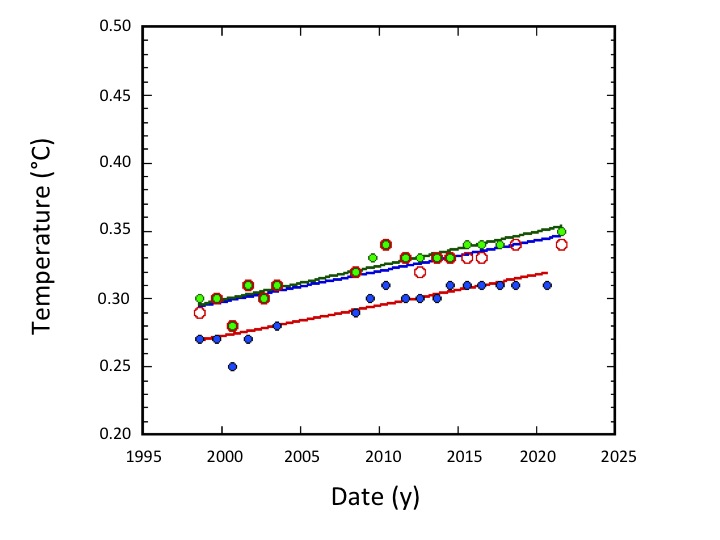


Fig. S1C. Temperature change of bottom waters of the Japan Basin (St. 6: green closed circles, St. 7: red open circles, St. 8: blue closed circles)


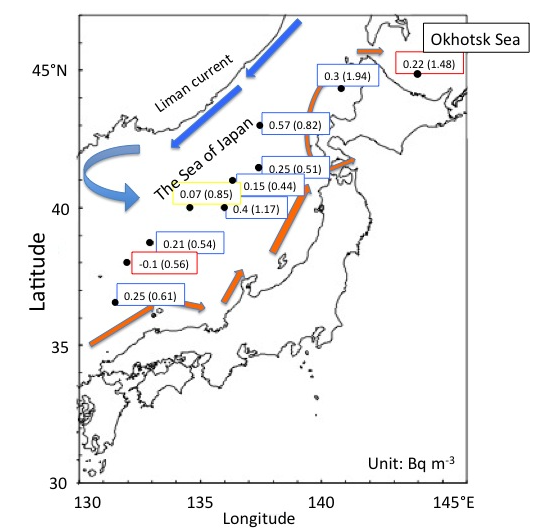


Fig. S2. Geographic distribution of increase rate of ^137^Cs in the overlying water in 2011. Increase rate in 2011 from a mean value from previous three years (observed ^137^Cs activity concentration in 2011). Blue square: > two sigma; Yellow square: <2 sigma; Red square: < 1 sigma. For the station numbers see Fig. 1.

**Tables**

Table S1. Location of sampling sites in the Sea of Japan (SOJ) and Okhotsk Sea (OS).

| Station | Latitude  (N) | Longitude  € | Depth  (m) | LN^a^ | WM^b^ | OWN^c^ |
| --- | --- | --- | --- | --- | --- | --- |
| 1 | 36.59 | 143.50 | 1981 | TB/UB | JSPMW | MDOW |
| 2 | 37.00 | 131.00 | 1680 | TB/UB | JSPW | MDOW |
| 3 | 38.72 | 132.93 | 2851 | TB/UB | BW | DOW |
| 4 | 40.00 | 134.56 | 1309 | YR | JSPW | MDOW |
| 5 | 40.00 | 136.00 | 1433 | YR | JSPW | MDOW |
| 6 | 41.00 | 136.34 | 3363 | JB | BW | DOW |
| 7 | 41.45 | 137.43 | 3635 | JB | BW | DOW |
| 8 | 43.01 | 137.49 | 3674 | JB | BW | DOW |
| 9 | 4434 | 140.82 | 253 |  |  | SOW |
| 10 | 44.80 | 144.00 | 186 | OS |  | SOW |

^a^LN (Location name) - TB/UB: Tsushima Basin/Ulleung Basin; YR: Yamato Ridge, JB: Japan Basin, OS: Okhotsk Sea.

^b^WM (Water mass) - JSPW: Japan Sea Proper Water; BW: Bottom Water.

^c^OWN (Overlying water name) - SOW: shallow overlying water; MDOW: middle depth overlying water; DOW: deep overlying water.

Table S2A. Sampling results observed for ten stations in the SOJ and OS in each year. Open circles: overlying water and sediment, triangles: only overlying water (sediment: less than detection limit), closed circles: only sediment, blank: no observation.

____________________________________________________________________________________________________________________________

Year Sampling station number

|  |  | 1 | 2 | 3 | 4 | 5 | 6 | 7 | 8 | 9 | 10 |
| --- | --- | --- | --- | --- | --- | --- | --- | --- | --- | --- | --- |
|  |  |  |  |  |  |  |  |  |  |  |  |
| 1998 |  | ○ | ○ | ○ | ○ | ○ | ○ | ○ | ○ | ○ | ○ |
| 1999 |  | ○ | ○ | ○ | ○ | ○ | ○ | ○ | ○ | ○ | ○ |
| 2000 |  | ○ | ● | ○ | ○ | ○ | ○ | ○ | ○ | ○ | ○ |
| 2001 |  | ○ | ○ | ○ | ○ | ○ | ○ | △ | △ | ● | ○ |
| 2002 |  | ○ | ○ | ○ | ○ | ○ | ○ | △ | △ | ○ | ○ |
| 2003 |  | ○ | ○ | ○ | ○ | ○ | ○ | ○ | ○ | ○ | ○ |
| 2004 |  | ○ |  |  | ○ | ○ |  |  |  | ○ | ○ |
| 2005 |  | ○ |  |  | ○ | ○ |  |  |  | ○ | ○ |
| 2006 |  | ○ | ○ | ○ | ○ | ○ |  |  |  | ○ | ○ |
| 2007 |  | ○ | ○ | △ | ○ | ○ |  |  |  | ○ | ○ |
| 2008 |  | ○ | ○ | △ | ○ | ○ | ○ | ○ | △ | ○ | ○ |
| 2009 |  | ○ | ○ | ○ | ○ | ○ | ○ |  | △ | ○ | ○ |
| 2010 |  | ○ | ○ | △ | ○ | ○ | ○ | ○ | △ | ○ | ○ |
| 2011 |  | ○ | ○ | ○ | ○ | ○ | ○ | ○ | ○ | ○ | ○ |
| 2012 |  |  |  |  | ○ | ○ | ○ | ○ | ○ | ○ | ○ |
| 2013 |  |  |  |  | ○ | ○ | ○ | ○ | ○ | ○ | ○ |
| 2014 |  |  |  |  | ○ |  | ○ | ○ | ○ | ○ |  |
| 2015 |  |  |  |  | ○ | ○ | ○ | ○ | ○ | ○ | ○ |
| 2016 |  |  |  |  | ○ | ○ | ○ | ○ | ○ | ○ | ○ |
| 2017 |  |  |  |  | ○ |  | ○ |  | △ | ○ |  |
| 2018 |  |  |  |  | ○ |  | ○ | ○ | ○ | ○ | ○ |
| 2019 |  |  |  |  |  |  |  |  |  |  |  |
| 2020 |  |  |  |  |  | ○ | ○ |  |  |  |  |
| 2021 |  |  |  |  | ○ | ○ |  | ○ | △ | ○ | ○ |

The sampling of sediments in Japan Basin (> 3000 m) during the period of 2004 – 2007 could not be conducted due to wire problems.

The sampling of three sites (St. 1, 2 and 3) has ceased after 2012 because of political issues.

Table S2B. Water depth (WD: m) and the overlying water layer depth (LD: m).

____________________________________________________________________________

St. 1 St. 2 St. 3 St. 4 St. 5

Year WD LD WD LD WD LD WD LD WD LD

______________________________________________________________________________

| 1998 |  | 1967 | 1917 | 1623 | 1573 | 2805 | 2755 | 1293 | 1243 | 1408 | 1358 |
| --- | --- | --- | --- | --- | --- | --- | --- | --- | --- | --- | --- |
| 1999 |  | 1926 | 1876 | 1579 | 1529 | 2780 | 2730 | 1232 | 1182 | 1386 | 1329 |
| 2000 |  | 1976 | 1926 | 1750 | 684 | 2849 | 2799 | 1307 | 1257 | 1422 | 1372 |
| 2001 |  | 1996 | 1889 | 1665 | 1570 | 2877 | 2764 | 1300 | 1155 | 1423 | 1335 |
| 2002 |  | 1992 | 1898 | 1700 | 1653 | 2875 | 2765 | 1305 | 1228 | 1450 | 1350 |
| 2003 |  | 2002 | 1913 | 1760 | 1665 | 2871 | 2798 | 1319 | 1231 | 1431 | 1365 |
| 2004 |  | 1993 | 1942 | 1284 | 1138 |  |  | 1291 | 1278 | 1432 | 1379 |
| 2005 |  | 1976 | 1903 |  |  |  |  | 1286 | 1220 | 1433 | 1371 |
| 2006 |  | 1980 | 1894 | 1700 | 1687 | 2860 | 2781 | 1311 | 1246 | 1482 | 1416 |
| 2007 |  | 1987 | 1919 | 1701 | 1645 | 2869 | 2789 | 1307 | 1247 | 1457 | 1407 |
| 2008 |  | 1984 | 1903 | 1694 | 1607 | 2858 | 2770 | 1280 | 1219 | 1413 | 1351 |
| 2009 |  | 1991 | 1889 | 1660 | 1574 | 2872 | 2766 | 1280 | 1231 | 1418 | 1358 |
| 2010 |  | 1973 | 1900 | 1706 | 1629 | 2852 | 2773 | 1326 | 1209 | 1430 | 1348 |
| 2011 |  | 1991 | 1909 | 1671 | 1574 | 2809 | 2724 | 1274 | 1237 | 1408 | 1327 |
| 2012 |  |  |  |  |  |  |  | 1330 | 1222 | 1446 | 1345 |
| 2013 |  |  |  |  |  |  |  | 1290 | 1210 | 1423 | 1346 |
| 2014 |  |  |  |  |  |  |  | 1297 | 1224 |  |  |
| 2015 |  |  |  |  |  |  |  | 1279 | 1229 | 1377 | 1327 |
| 2016 |  |  |  |  |  |  |  | 1370 | 1224 | 1440 | 1345 |
| 2017 |  |  |  |  |  |  |  | 1314 | 1219 |  |  |
| 2018 |  |  |  |  |  |  |  | 1325 | 1244 |  |  |
| 2019 |  |  |  |  |  |  |  | 1261 | 1211 |  |  |
| 2020 |  |  |  |  |  |  |  |  |  | 1396 | 1346 |
| 2021 |  |  |  |  |  |  |  | 1267 | 1210 | 1405 | 1327 |

_____________________________________________________________________________

___________________________________________________________________________

St. 6 St. 7 St. 8 St. 9 St. 10

Year WD LD WD LD WD LD WD LD WD LD

_____________________________________________________________________________

| 1998 |  | 3327 | 3277 | 3598 | 3548 | 3636 | 3586 | 254 | 237 | 181 | 174 |
| --- | --- | --- | --- | --- | --- | --- | --- | --- | --- | --- | --- |
| 1999 |  | 3311 | 3261 | 3587 | 3537 | 3618 | 3568 | 254 | 204 | 183 | 132 |
| 2000 |  | 3400 | 3350 | 3642 | 3635 | 3685 | 3635 | 258 | 225 | 187 | 147 |
| 2001 |  | 3390 | 3279 | 3660 | 3553 | 3690 | 3580 | 254 | 254 | 190 | 178 |
| 2002 |  | 3409 | 3293 | 3661 | 3558 | 3702 | 3583 | 253 | 245 | 186 | 175 |
| 2003 |  | 3390 | 3326 | 3666 | 3616 | 3692 | 3641 | 253 | 203 | 181 | 132 |
| 2004 |  |  |  |  |  |  |  | 251 | 198 | 186 | 129 |
| 2005 |  |  |  |  |  |  |  | 254 | 200 | 183 | 129 |
| 2006 |  |  |  |  |  |  |  | 243 | 201 | 186 | 134 |
| 2007 |  |  |  |  |  |  |  | 258 | 206 | 185 | 136 |
| 2008 |  | 3351 | 3279 | 3630 | 3561 | 3667 | 3588 | 259 | 208 | 183 | 150 |
| 2009 |  | 3391 | 3280 | 3666 | 3544 | 3692 | 3579 | 260 | 201 | 185 | 132 |
| 2010 |  | 3387 | 3284 | 3666 | 3574 | 3697 | 3601 | 258 | 201 | 188 | 134 |
| 2011 |  | 3326 | 3275 | 3587 | 3540 | 3682 | 3578 | 257 | 198 | 186 | 137 |
| 2012 |  | 3387 | 3288 | 3659 | 3555 | 3692 | 3591 | 265 | 201 | 186 | 126 |
| 2013 |  | 3368 | 3268 | 3683 | 3552 | 3686 | 3579 | 229 | 178 | 190 | 137 |
| 2014 |  | 3362 | 3279 | 3625 | 3564 | 3657 | 3571 | 253 | 196 |  |  |
| 2015 |  | 3342 | 3292 | 3597 | 3547 | 3631 | 3581 | 255 | 205 | 186 | 136 |
| 2016 |  | 3386 | 3292 | 3664 | 3539 | 3690 | 3582 | 260 | 183 | 188 | 137 |
| 2017 |  | 3400 | 3298 |  |  | 3710 | 3598 | 253 | 194 |  |  |
| 2018 |  | 3380 | 3277 | 3647 | 3556 | 3674 | 3587 | 253 | 200 | 188 | 136 |
| 2019 |  | 3329 | 3279 |  |  | 3615 | 3565 |  |  |  |  |
| 2020 |  | 3313 | 3263 |  |  |  |  |  |  |  |  |
| 2021 |  |  |  | 3635 | 3549 | 3691 | 3585 | 253 | 202 | 188 | 137 |

__________________________________________________________________

Table S3. Activity concentrations of ^137^Cs in overlying waters of the SOJ and OS.

_____________________________________________________________________________________________________________________

Sampling station Sample No. Activity concentration (Bq m^-3^)

Range Mean SD

| 1 |  | 14 |  | 0.25 | - | 0.61 | 0.35 | 0.09 |
| --- | --- | --- | --- | --- | --- | --- | --- | --- |
| 2 |  | 11 |  | 0.34 | - | 0.73 | 0.58 | 0.1 |
| 3 |  | 12 |  | 0.23 | - | 0.54 | 0.33 | 0.08 |
| 4 |  | 23 |  | 0.59 | - | 1.15 | 0.83 | 0.18 |
| 5 |  | 19 |  | 0.44 | - | 1.17 | 0.76 | 0.16 |
| 6 |  | 18 |  | 0.11 | - | 0.6 | 0.34 | 0.11 |
| 7 |  | 16 |  | 0.13 | - | 0.51 | 0.29 | 0.1 |
| 8 |  | 16 |  | 0.14 | - | 0.82 | 0.35 | 0.18 |
| 9 |  | 21 |  | 1.5 | - | 2.3 | 1.83 | 0.26 |
| 10 |  | 20 |  | 0.71 | - | 2.31 | 1.32 | 0.34 |

____________________________________________________________________________________________

Table S4. ^137^Cs activity concentrations in overlying water on January 1998, apparent change rates and physical change rates of ^137^Cs in overlying waters.

A: pre-Fukushima era. Sampling period: 1998 - 2010

_______________________________________________________________________________________________________________________________

Station No. Depth (m) C_137Cs,OW,o_ (Bq m^-3^) k_aw_ (y^-1^) k_pw_ (y^-1^)

| 1 |  | 1981 |  | 0.30 | ± | 0.03 |  | 0.016 | ± | 0.011 |  | 0.039 | ± | 0.011 |
| --- | --- | --- | --- | --- | --- | --- | --- | --- | --- | --- | --- | --- | --- | --- |
| 2 |  | 1680 |  | 0.60 | ± | 0.07 |  | -0.005 | ± | 0.014 |  | 0.018 | ± | 0.014 |
| 3 |  | 2851 |  | 0.31 | ± | 0.03 |  | 0.004 | ± | 0.01 |  | 0.027 | ± | 0.01 |
| 4 |  | 1309 |  | 0.99 | ± | 0.07 |  | -0.022 | ± | 0.014 |  | 0.001 | ± | 0.014 |
| 5 |  | 1433 |  | 0.68 | ± | 0.07 |  | 0.004 | ± | 0.007 |  | 0.027 | ± | 0.007 |
| 6 |  | 3373 |  | 0.25 | ± | 0.05 |  | 0.017 | ± | 0.02 |  | 0.040 | ± | 0.02 |
| 7 |  | 3635 |  | 0.19 | ± | 0.03 |  | 0.029 | ± | 0.02 |  | 0.052 | ± | 0.02 |
| 8 |  | 3674 |  | 0.20 | ± | 0.03 |  | 0.021 | ± | 0.02 |  | 0.044 | ± | 0.02 |
| 9 |  | 235 |  | 2.32 | ± | 0.05 |  | -0.031 | ± | 0.003 |  | -0.008 | ± | 0.003 |
| 10 |  | 186 |  | 1.77 | ± | 0.19 |  | -0.038 | ± | 0.017 |  | -0.015 | ± | 0.017 |

______________________________________________________________________________________________

B: Full period including post Fukushima. St. 1 - 3: 1998 – 2011, St. 4, 5, 8, 7, 9: 1998 – 2021, St. 6: 1998 – 2020, St. 8: 1998 - 2017

_______________________________________________________________________________________________________________________________

Station No. Depth (m) C_137Cs,OW,o_ (Bq m^-3^) k_aw_ (y^-1^) k_pw_ (y^-1^)

| 1 |  | 1981 |  | 0.26 | ± | 0.04 |  | 0.038 | ± | 0.014 |  | 0.061 | ± | 0.014 |
| --- | --- | --- | --- | --- | --- | --- | --- | --- | --- | --- | --- | --- | --- | --- |
| 2 |  | 1680 |  | 0.60 | ± | 0.06 |  | -0.005 | ± | 0.012 |  | 0.018 | ± | 0.012 |
| 3 |  | 2851 |  | 0.21 | ± | 0.04 |  | 0.028 | ± | 0.01 |  | 0.051 | ± | 0.01 |
| 4 |  | 1309 |  | 0.91 | ± | 0.07 |  | -0.008 | ± | 0.006 |  | 0.015 | ± | 0.0062 |
| 5 |  | 1433 |  | 0.66 | ± | 0.06 |  | 0.013 | ± | 0.007 |  | 0.036 | ± | 0.007 |
| 6 |  | 3373 |  | 0.23 | ± | 0.04 |  | 0.030 | ± | 0.011 |  | 0.053 | ± | 0.011 |
| 7 |  | 3635 |  | 0.28 | ± | 0.04 |  | 0.024 | ± | 0.016 |  | 0.047 | ± | 0.016 |
| 8 |  | 3674 |  | 0.20 | ± | 0.04 |  | 0.05 | ± | 0.02 |  | 0.073 | ± | 0.02 |
| 9 |  | 235 |  | 2.17 | ± | 0.09 |  | -0.015 | ± | 0.004 |  | 0.008 | ± | 0.004 |
| 10 |  | 186 |  | 1.70 | ± | 0.14 |  | -0.026 | ± | 0.008 |  | -0.003 | ± | 0.008 |

_____________________________________________________________________________________________

Table S5. Activity concentrations of ^137^Cs in sediments of the SOJ and OS.

_________________________________________________________________________

Sampling station Sample No. Activity concentration (Bq kg^-1^)

Range Mean SD

| 1 |  | 14 |  | 1 | - | 4.8 | 2.38 | 0.97 |
| --- | --- | --- | --- | --- | --- | --- | --- | --- |
| 2 |  | 12 |  | 0.91 | - | 3.23 | 2.17 | 0.61 |
| 3 |  | 9 |  | 0.01 | - | 0.54 | 0.22 | 0.16 |
| 4 |  | 22 |  | 0.58 | - | 2.8 | 2.14 | 0.54 |
| 5 |  | 19 |  | 1.1 | - | 3.2 | 2.1 | 0.53 |
| 6 |  | 18 |  | 1.1 | - | 6 | 3.14 | 1.2 |
| 7 |  | 16 |  | 0.18 | - | 3.7 | 2.34 | 0.89 |
| 8 |  | 10 |  | 0.02 | - | 0.35 | 0.14 | 0.12 |
| 9 |  | 22 |  | 1.3 | - | 3.35 | 2.47 | 0.57 |
| 10 |  | 20 |  | 1.2 | - | 2.9 | 1.87 | 0.42 |

____________________________________________________________________________________________

Table S6. ^137^Cs activity concentrations in sediments on January 1998, apparent change rates and physical change rates of ^137^Cs in sediments.

A. Pre-Fukushima era. Sampling period: 1998 – 2010.

_______________________________________________________________________________________________________________________________

Station No. Depth (m) C_137Cs,SS,o_ (Bq kg^-1^) k_aS_ (y^-1^) k_pS_ (y^-1^)

| 1 |  | 1981 |  | 1.84 | ± | 0.49 |  | 0.033 | ± | 0.032 |  | 0.066 | ± | 0.032 |
| --- | --- | --- | --- | --- | --- | --- | --- | --- | --- | --- | --- | --- | --- | --- |
| 2 |  | 1680 |  | 2.29 | ± | 0.33 |  | -0.016 | ± | 0.020 |  | 0.007 | ± | 0.02 |
| 3 |  | 2851 |  | - |  |  |  | - |  |  |  | - |  |  |
| 4 |  | 1309 |  | 2.09 | ± | 0.28 |  | 0.011 | ± | 0.017 |  | 0.034 | ± | 0.017 |
| 5 |  | 1433 |  | 2.23 | ± | 0.30 |  | -0.001 | ± | 0.018 |  | 0.022 | ± | 0.018 |
| 6 |  | 3373 |  | 3.48 | ± | 0.83 |  | -0.003 | ± | 0.033 |  | 0.020 | ± | 0.033 |
| 7 |  | 3635 |  | 2.32 | ± | 0.57 |  | -0.038 | ± | 0.046 |  | -0.015 | ± | 0.046 |
| 8 |  | 3674 |  |  |  |  |  |  |  |  |  |  |  |  |
| 9 |  | 235 |  | 3.08 | ± | 0.26 |  | -0.025 | ± | 0.012 |  | -0.002 | ± | 0.012 |
| 10 |  | 186 |  | 2.51 | ± | 0.16 |  | -0.033 | ± | 0.010 |  | -0.010 | ± | 0.010 |

______________________________________________________________________________________________

B: Full period including post Fukushima. St. 1 - 3: 1998 – 2011, St. 4, 5, 8, 7, 9: 1998 – 2021, St. 6: 1998 – 2020, St. 8: 1998 – 2017

_______________________________________________________________________________________________________________________________

Station No. Depth (m) C_137Cs,SS,o_ (Bq kg^-1^) k_aS_ (y^-1^) k_pS_ (y^-1^)

| 1 |  | 1981 |  | 1.74 | ± | 0.44 |  | 0.039 | ± | 0.026 |  | 0.062 | ± | 0.026 |
| --- | --- | --- | --- | --- | --- | --- | --- | --- | --- | --- | --- | --- | --- | --- |
| 2 |  | 1680 |  | 2.11 | ± | 0.35 |  | 0.004 | ± | 0.019 |  | 0.027 | ± | 0.002 |
| 3 |  | 2851 |  |  | - |  |  |  | - |  |  |  | - |  |
| 4 |  | 1309 |  | 2.32 | ± | 0.24 |  | -0.007 | ± | 0.008 |  | 0.016 | ± | 0.008 |
| 5 |  | 1433 |  | 2.41 | ± | 0.24 |  | -0.015 | ± | 0.009 |  | 0.008 | ± | 0.009 |
| 6 |  | 3373 |  | 1.9 | ± | 0.38 |  | -0.014 | ± | 0.013 |  | 0.009 | ± | 0.013 |
| 7 |  | 3635 |  | 3.67 | ± | 0.58 |  | 0.017 | ± | 0.013 |  | 0.04 | ± | 0.013 |
| 8 |  | 3674 |  |  | - |  |  | - |  |  |  | - |  |  |
| 9 |  | 235 |  | 3.01 | ± | 0.24 |  | -0.02 | ± | 0.007 |  | 0.003 | ± | 0.007 |
| 10 |  | 186 |  | 2.39 | ± | 0.15 |  | -0.025 | ± | 0.006 |  | -0.002 | ± | 0.006 |

______________________________________________________________________________________________

Table S7. Practical partition coefficients of ^137^Cs between overlying water and sediment.

_______________________________________________________________

Sampling station Sample No. Practical partition coefficient (×10^3^ L kg^-1^)

Range Mean SD

| 1 |  | 14 |  | 3.3 | - | 14.5 | 7 | 3.1 |
| --- | --- | --- | --- | --- | --- | --- | --- | --- |
| 2 |  | 12 |  | 1.6 | - | 6.2 | 3.9 | 1.4 |
| 3 |  | 9 |  | 0.31 | - | 1.45 | 0.66 | 0.46 |
| 4 |  | 22 |  | 0.88 | - | 4.51 | 2.61 | 0.92 |
| 5 |  | 19 |  | 1.51 | - | 4.21 | 2.88 | 0.88 |
| 6 |  | 18 |  | 3 | - | 47.2 | 11.5 | 10 |
| 7 |  | 16 |  | 0.69 | - | 14.9 | 8.6 | 3.5 |
| 8 |  | 10 |  | 0.12 | - | 0.85 | 0.34 | 0.26 |
| 9 |  | 22 |  | 0.76 | - | 2 | 1.33 | 0.34 |
| 10 |  | 20 |  | 0.86 | - | 2.1 | 1.41 | 0.38 |

_____________________________________________________________________________________________

Table S8. Practical partition coefficients in 1998 and apparent change rates of K_d_ values.

1. Pre-Fukushima era. Sampling period: 1998 – 2010

_____________________________________________________________________________________________

Station No. Depth (m) K_d,o_ (×10^3^ L kg^-1^) k_kd_ (y^-1^) Correlation factor

| 1 |  | 1981 |  | 7.14 | ± | 1.86 |  | -0.0067 | ± | 0.034 |  | 0.009 |
| --- | --- | --- | --- | --- | --- | --- | --- | --- | --- | --- | --- | --- |
| 2 |  | 1680 |  | 4.06 | ± | 0.86 |  | -0.015 | ± | 0.028 |  | 0.190 |
| 3 |  | 2851 |  |  | - |  |  | - |  |  |  | - |
| 4 |  | 1309 |  | 2.25 | ± | 0.47 |  | 0.029 | ± | 0.025 |  | 0.328 |
| 5 |  | 1433 |  | 3.30 | ± | 0.42 |  | 0.0027 | ± | 0.017 |  | 0.047 |
| 6 |  | 3373 |  | 23.5 | ± | 9.5 |  | -0.087 | ± | 0.086 |  | 0.383 |
| 7 |  | 3635 |  | 12.4 | ± | 2.7 |  | -0.060 | ± | 0.045 |  | 0.564 |
| 8 |  | 3674 |  |  | - |  |  |  | - |  |  | - |
| 9 |  | 235 |  | 1.30 | ± | 0.13 |  | 0.0082 | ± | 0.013 |  | 0.047 |
| 10 |  | 186 |  | 1.54 | ± | 0.22 |  | 0.0007 | ± | 0.019 |  | 0.011 |

_____________________________________________________________________________________________

B: Full period including post Fukushima. St. 1 - 3: 1998 – 2011, St. 4, 5, 8, 7, 9: 1998 – 2021, St. 6: 1998 – 2020, St. 8: 1998 – 2017

_____________________________________________________________________________________________

Station No. Depth (m) K_d,o_ (×10^3^ L kg^-1^) k_kd_ (y^-1^) Correlation factor

| 1 |  | 1981 |  | 7.47 | ± | 1.78 |  | -0.0092 | ± | 0.03 |  | 0.079 |
| --- | --- | --- | --- | --- | --- | --- | --- | --- | --- | --- | --- | --- |
| 2 |  | 1680 |  | 3.69 | ± | 0.86 |  | 0.006 | ± | 0.026 |  | 0.072 |
| 3 |  | 2851 |  |  | - |  |  |  | - |  |  | - |
| 4 |  | 1309 |  | 2.71 | ± | 0.4 |  | -0.0028 | ± | 0.012 |  | 0.056 |
| 5 |  | 1433 |  | 3.65 | ± | 0.39 |  | -0.025 | ± | 0.011 |  | 0.513 |
| 6 |  | 3373 |  | 22.1 | ± | 5.3 |  | -0.067 | ± | 0.029 |  | 0.525 |
| 7 |  | 3635 |  | 10.7 | ± | 1.7 |  | -0.021 | ± | 0.014 |  | 0.373 |
| 8 |  | 3674 |  | - |  |  |  | - |  |  |  | - |
| 9 |  | 235 |  | 1.36 | ± | 0.15 |  | 0.0023 | ± | 0.009 |  | 0.06 |
| 10 |  | 186 |  | 1.45 | ± | 0.2 |  | -0.001 | ± | 0.012 |  | 0.019 |

____________________________________________________________________________________________

**Distances from the seafloor to the sampling depth**

The distances between the overlying layer and the seafloor surface ranged from 44 m to 86 m (given as mean values for the stations, Table S9). In general, variability of the distance from the seafloor to the sampling depth is considered to cause variability of the ^137^Cs concentration in seawater, which affects the evaluation of K_d_ values. In this case, vertical distributions of ^137^Cs activity concentrations in the water column are important to evaluate the effects of variation of the sampling distance. The ^137^Cs levels in SOJ water columns are primarily governed by vertical diffusion process.^1^ Since more than 35 years spent from ^137^Cs injection due to global fallout, the vertical profiles of ^137^Cs show a thick surface homogeneous layer, a relatively large gradient from 500 to 2000 m, and a homogeneous layer in depths > 2000 m. The K_d_ values in shallow layers (St. 9 and St. 10) and in bottom water (> 2500 m depth; St.3, St.6, St.7 and St.8) are insensitive for changes in the distance from the seafloor to the sampling depth, while K_d_ of some sites in JSPW (1000 – 2000 m depth; St. 1, St. 2, St. 4, St.5) may be affected by distance variability.

References

1. Hirose, K., Povinec, P.P. ^90^Sr and ^137^Cs as tracers of oceanic eddies in the Sea of Japan/East Sea. J. Environ. Radioact. 216, 106179 (2020).

Table S9. Distances from the seafloor to the sampling depth (unit: m).

___________________________________________________________

Sampling site Min Max Mean Median Standard dev.

___________________________________________________________

St. 1 50 107 75 77 20

St. 2 13 146 75 82 34

St. 3 50 113 80 80 22

St. 4 13 146 73 65 33

St. 5 50 101 69 64 18

St. 6 50 116 79 83 26

St. 7 7 131 80 86 34

St. 8 50 119 86 96 27

St. 9 0 77 48 52 18

St. 10 7 60 44 51 16

______________________________________________________

**Sediment properties**

Deep-sea sediments (>500 m water depth) generally comprise of lithogenous materials, typically Aeolian dust (Asian dust) and biogenic products, in which most of the lithogenous inorganic materials were clay-sized components (< 2 μm)^1^. In the SOJ, deep-sea sediments are characterized as terrigenous sediments comprising of mud (or clay and ooze; Table S10), which are composed of fine particles instead of sand. Major chemical compositions of deep-sea sediments are lithogenous and biogenous (calcareous and/or siliceous), in which in general, deep-sea carbonates are impoverished in most trace elements comparing with deep-sea clay (ex. deep-sea clay: Cs 5 μg g^-1^, calcareous: Cs 0.4 μg g^-1^) except Sr.

^137^Cs activity concentrations in pelagic sediments (> 2500 m water depth) of the Japan Basin varied largely spatiotemporally, and especially showed low ^137^Cs levels. A possible chemical cause of large variability may be in heterogeneous distribution of lithogenous and calcareous components of sediments. Another cause may be in physical perturbation accompanied with relocation and resuspension of ^137^Cs-enriched particles at the sediment surface due to turbulences from instability of ocean floor current, as well as due to earthquakes^2^.

1. Chester, R. *Marine Geochemistry*. 2^nd^ Ed. (Blackwell Science, 2003).
2. Nakajima, T., Kanai, Y. Sedimentary features of semiturbidities triggered by the 1983 and older historical earthquakes in the eastern margin of the Japan Sea. *Sedimentary Geol*. **135**, 1–19 (2000).

Table S10. Morphology of sediments.

_________________________________________________________________________

Year Sampling station number

|  |  | 1 | 2 | 3 | 4 | 5 | 6 | 7 | 8 | 9 | 10 |
| --- | --- | --- | --- | --- | --- | --- | --- | --- | --- | --- | --- |
|  |  |  |  |  |  |  |  |  |  |  |  |
| 2014 |  |  |  |  | M |  | M | M | M | M |  |
| 2015 |  |  |  |  | M | M | M | M | M | M | M |
| 2016 |  |  |  |  | M | M | M | M | M | M | M |
| 2017 |  |  |  |  | M |  | M |  | M | M |  |
| 2018 |  |  |  |  | Oz |  | Oz,Cy | Oz | Oz | Cy | Cy |
| 2019 |  |  |  |  |  |  |  |  |  |  |  |
| 2020 |  |  |  |  |  | M | Oz |  |  |  |  |
| 2021 |  |  |  |  | M | M,Oz |  | Oz | M,Oz | M,Oz | M |

______________________________________________________________________________________________M: Mud, Oz: Ooze, Cy: Clay

There was no description of sediment properties before 2014.
